# Distinguishing Common Digital Phenotyping and Self-Report Parameters for Monitoring and Predicting Depression: Scoping Review

**DOI:** 10.2196/70840

**Published:** 2026-03-02

**Authors:** Lisa Busshart, Milica Petrovic, Rebeka Amin, Ulrich Hegerl

**Affiliations:** 1 Department of Psychiatry, Psychosomatics and Psychotherapy, University Hospital Frankfurt, Research Center of German Foundation for Depression and Suicide Prevention, Heinrich-Hoffmann-Str. 10, Frankfurt am Main, 60528, Germany, 49 162 9464667; 2 Department for Psychiatry, Psychosomatics and Psychotherapy, Goethe University Frankfurt, Goethe Research Professorship, Frankfurt am Main, Germany

**Keywords:** depression, digital phenotyping, sensor data, objective data, self-monitoring

## Abstract

**Background:**

Digital health interventions incorporating self-management strategies are increasingly used to support individuals in managing depression. These interventions often leverage self-monitoring and passive sensor-based data collection to provide personalized feedback, guiding behavioral change. With the proliferation of smartphones and wearable devices, there is growing potential to continuously collect behavioral and physiological data. However, a major limitation in the field is the lack of consolidated evidence on which specific parameters are most useful for monitoring and predicting depression-related outcomes.

**Objective:**

This scoping review aims to identify and synthesize common digital phenotyping and self-report parameters for monitoring and predicting depression. Specifically, it addresses the methodological and knowledge gap concerning which types of sensor-based and self-reported data are most frequently used and which demonstrate predictive value in tracking changes in depressive symptoms across digital platforms.

**Methods:**

A literature search was conducted across 4 databases, including PubMed, Embase, Cochrane Library, and the Web of Science Core Collection. Articles published between January 1, 2021, and November 26, 2025, were included. Eligible studies included adults (≥18 years) with depression confirmed through validated clinical measures and using digital approaches that collected passive sensor data, self-reports, or both. Studies focusing on comorbid disorders, nondigital interventions, or not reporting depression-related outcomes were excluded. The PRISMA-ScR (Preferred Reporting Items for Systematic Reviews and Meta-Analyses Extension for Scoping Reviews) guidelines were followed, and a 5-stage methodological framework for scoping reviews was implemented. Quality assessment was performed using the Downs and Black Instrument and the Mixed Methods Appraisal Tool (MMAT).

**Results:**

Nineteen studies were included, comprising a total of 85,193 participants. Most studies used smartphone- or wearable-based tools, with passive sensing as the predominant data source and Patient Health Questionnaire-9 (PHQ-9) as the most commonly used depression measure. Five overarching parameter categories were identified: (1) physical activity and location, (2) behavioral patterns, (3) physiological signals, (4) sleep indicators, and (5) sociability and self-reported assessments. Within these categories, 11 metrics, including step count, heart rate variability, sleep duration and mood self-ratings, were most frequently reported. Most studies used a multimodal digital phenotyping approach, integrating passive sensor-derived data with active user-reported input, enabling more individualized symptom monitoring over time.

**Conclusions:**

This scoping review provides a novel synthesis of common digital parameters used across diverse tools for monitoring and predicting depression, moving beyond tool- or modality-specific perspectives adopted in prior reviews. Unlike existing reviews focusing on individual sensing modalities, prediction methods, or intervention effectiveness, this review maps shared parameters across observational, predictive, and interventional studies. By identifying convergent digital markers, the review supports comparability across studies and informs future model development. These findings have practical implications for the design of scalable digital mental health tools and for translating digital phenotyping into real-world clinical and self-management contexts.

## Introduction 

Advancements in personal digital devices and sensor technologies have enabled continuous and unobtrusive data collection from individuals in real-world settings, expanding the possibilities in health care data management [1]. This has given rise to digital phenotyping, defined as the moment-by-moment quantification of individual-level behavior and aspects of physiology using data from personal smartphones and wearable devices of users [2].

The sensors commonly embedded in these devices can be broadly categorized into (1) motion sensors, (2) location sensors, and (3) environment sensors [3]. More specifically, smartphone-based sensors include linear acceleration, rotation vector, humidity, orientation, gravity, magnetic field, accelerometer, ambient temperature, gyroscope, light, pressure, microphone, proximity, and others [4]. Each device uses multiple sensors working together to provide optimal output for the user. For instance, environmental sensors, including humidity, illumination intensity, surrounding pressure, and temperature sensors enable automatic smartphone screen brightness adjustment or calculation of the dew point for the day [3]. Similarly, the geomagnetic sensor and accelerometer show user-relative location to the North Pole, providing basic data for location sensors. Motion sensors assess acceleration, speed of revolution, gravity, curl vector values, and drift, allowing tracking of multiple motions of the user, such as car direction (eg, navigation application), steps walked (eg, fitness or exercise mobile applications), and speed of movement (eg, user car speed) [3,5].

Unobtrusive tracking of health data relying on physiological parameters such as heart rate, step count, sleep, and others commonly occurs by using sensor fusion (ie, putting multiple signals together) [6]. For instance, biosensors can track physical parameters such as heart rate [7], while location sensors can disclose patient activity and potentially help infer how often outdoor activities occur, which is particularly important for individuals living with mood disorders such as depression [8]. Social patterns can be monitored through the aggregated amount of mobile phone calls and textual messages exchanged with others, pointing to social activity or potential social withdrawal, often serving as an indicator of a depressive episode [9]. Finally, sensor fusion can be used by combining multiple sensors such as accelerometer, ambient light, battery level, microphone, phone lock status, phone unlock status, and screen status for the physiological parameter of sleep that has a significant role in indicating depressive episodes [10].

Some of the earlier works that explored the field of digital phenotyping in mental health include a systematic review [11] of smartphone-based passive sensing, summarizing common data sources such as GPS, accelerometry, and phone usage metrics. Several research papers have outlined the promise of personal sensing approaches in psychiatry while also emphasizing challenges related to data interpretation and real-world implementation [12,13]. Early empirical support for the clinical relevance of smartphone-derived data has been demonstrated through significant correlations between sensor-based measures and depressive symptom severity [14].

Recent reviews have demonstrated growing interest in the use of passively collected data for depression monitoring, reflecting the rapid advancement of digital phenotyping approaches. For example, several studies have focused on specific technologies, such as smartphone-based sensing alone, or emphasized algorithm development for symptom detection rather than broader clinical integration [15-18]. Others have targeted a narrower set of parameters [19], which limits their scope for cross-study synthesis. In contrast, the present review aims to provide a comprehensive overview of the parameters most commonly used in digital interventions for depression, regardless of delivery platform, by synthesizing data across smartphone- and wearable-based sensing studies. This broader focus is intended to support standardization efforts and inform the development of more effective and clinically relevant digital mental health tools.

There is a considerable variability in the types of parameters used, the degree of personalization, and the delivery modes, making it difficult to compare studies or determine which features are most effective [19-22]. This lack of consistency makes it difficult to develop reliable digital health tools. Moreover, health practitioners and end users often lack clear guidance on which digital tools offer meaningful and reliable support.

To address such gaps, this review aims to map the landscape of passively collected parameters used in digital interventions for depression, with the goal of informing future research, standardization, and clinical application.

This review is guided by the following research questions:

1. What passively collected parameters are commonly used in digital interventions for depression to support longitudinal monitoring of depressive symptoms?
  - Which types of passively collected data are used?
  - Which parameters are most frequently used?
  - Which parameters have demonstrated predictive value for changes in symptom severity?

We define a parameter as any sensor-derived measure used to monitor or predict depression-related outcomes.

## Methods 

### Study Design and Framework

This scoping review was conducted in accordance with the 5-stage methodological framework for scoping reviews, including (1) identifying the research question, (2) identifying relevant studies, (3) study selection, (4) data charting and synthesis, and (5) collating, summarizing, and reporting results [23]. The data were systematically collected and reported by following the PRISMA-ScR (Preferred Reporting Items for Systematic Reviews and Meta-Analyses Extension for Scoping Reviews) guidelines (Checklist 1) [24].

This scoping review was not registered in PROSPERO (International Prospective Register of Systematic Reviews), as PROSPERO does not accept registrations for scoping review protocols at the time of this writing.

### Data Sources and Search Strategy

We conducted a comprehensive search of 4 electronic databases, including PubMed, Embase, Cochrane Library, and Web of Science Core Collection. Study registries were searched, including ClinicalTrials.gov and the World Health Organization International Clinical Trials Registry Platform (WHO ICTRP). The findings from these registries are categorized under “Others” in the PRISMA flow chart. No online or print sources were purposefully searched or browsed outside of the 4 electronic databases. No table of contents, conference proceedings, organizational websites, or print materials were hand-searched for this review. However, reference lists of included articles were also manually screened to identify additional eligible studies, which are categorized under “Other” in the PRISMA flow chart. No databases were searched simultaneously on a single platform, and no additional studies or data were sought by contacting study authors, experts, manufacturers, or others.

Studies published between January 1, 2021, and November 26, 2025, were included to reflect the recent advancements in digital phenotyping and mobile sensor technology in mental health. Our searches applied limits for publication date and language. All searches were last conducted on November 26, 2025 across all included databases, restricted to publications in English or German. These limits were used to ensure that the review captured the most current developments in digital interventions and digital phenotyping, as the technological landscape in this field evolves rapidly. No additional limits (eg, study design filters) were applied. The searches were updated manually by rerunning all database queries prior to final analysis. No automated alerts, citation tracking tools, or continuous updating services were used. The search strategy combined controlled vocabulary where applicable (eg, medical subject headings [MeSH] in PubMed) and free-text terms related to depression, digital health interventions, sensor data, and digital phenotyping. No published search filters were used for this review, and no search strategies from previous literature reviews were adapted or reused. All search strategies were developed manually using combinations of keywords and medical subject headings relevant to depression, digital phenotyping, and digital health interventions. The search strategy did not use any existing validated filters (eg, clinical study filters or methodological filters). No formal search peer review process was conducted for this review.

A summary of keyword combinations is provided in Table 1. For detailed search strategy see PRISMA-ScR Checklist 1 containing a detailed search strategy across databases.

**Table 1.** Search strategy with combined keyword terms and date range.

Database	Keywords combined	Date range
PubMed	“Depression” AND “digital health intervention” “Depression” AND “digital phenotyping” “Self-management” OR “Depression” AND “digital phenotyping” “Depression” AND “digital phenotyping” AND “passive intervention” “Depression” AND “sensor data”	January 1, 2021-November 26, 2025
EMBASE	“Depression,” “digital health intervention” “Depression,” “digital phenotyping” “Self-management” / “Depression,” “digital phenotyping” “Depression,” “digital phenotyping,” “passive intervention” “Depression,” “sensor data”	January 1, 2021-November 26, 2025
Cochrane Library	“Depression” AND “digital health intervention” “Depression” AND “digital phenotyping” “Self-management” OR “Depression” AND “digital phenotyping” “Depression” AND “digital phenotyping” AND “passive intervention” “Depression” AND “sensor data”	January 1, 2021-November 26, 2025
Web of Science Core Collection	“Depression,” “digital health intervention” “Depression,” “digital phenotyping” “Self-management” / “Depression,” “digital phenotyping” “Depression,” “digital phenotyping,” “passive intervention” “Depression,” “sensor data”	January 1, 2021-November 26, 2025

A total of 3243 records were identified during the study selection process, screened (n=1406), and reviewed for eligibility (n=134). After applying inclusion and exclusion criteria, a total of 19 studies were included in the final analysis. Reasons for exclusion included studies without digital interventions, nonclinical populations, and studies focused on comorbid conditions or unrelated mental health disorders.

The total number of records identified within each database and other information sources was 3243 (PubMed n=518; Cochrane Library n=1256; EMBASE n=791; Web of Science n=276; and Others n=402). Relevant records were identified from 4 databases (n=518) and clinical trial registers (n=2725). After duplicate removal, 1406 records were screened. Following full-text screening and exclusions, 19 studies were included in the final review. Common reasons for exclusion included lack of intervention focus (n=27), nonclinical populations (n=13), or nondigital approaches (n=9). Records retrieved from all databases and clinical trial registries were exported into Microsoft Excel and manually reviewed to identify and remove duplicates. Deduplication was performed by comparing study titles, authors, publication years, and digital object identifiers. No automated or software-assisted deduplication tools (eg, Covidence [Veritas Health Innovation] or EndNote [Clarivate]) were used.

### Eligibility Criteria

Studies including randomized controlled trials, observational studies, and mixed method primary studies were considered. Eligible studies include digital health interventions for adults (≥18 years) concretely addressing depression that is assessed primarily with clinical measures such as the Patient Health Questionnaire-9 (PHQ-9) or similar validated instruments. The population focus is mainly on participants that, at the time of study participation, were experiencing depression, noted with a clinical measurement tool and digital phenotyping methodology. Study reports on outcomes related to the assessment of digital phenotyping for depression or dedicate a substantial amount of work to such reporting. The timeframe for published studies was limited from January 1, 2021 to November 26, 2025 to capture the most recent advances in digital phenotyping and mobile sensor monitoring in mental health. This period marks a notable shift from early feasibility and pilot work to more sophisticated methods, including multisite validation, machine learning–based prediction models, and integration of wearables and smartphone sensors [25]. By focusing on this timeframe, we ensure that the review remains aligned with current technological capabilities and clinically relevant research directions.

### Study Selection

Title and abstract preselection was performed by 2 authors LB and MP. Screening questions included:

1. Does the study use a digital intervention for depression delivered via a digital tool (eg, mobile app, wearable, web-based platform)?
2. Does the study report on depression outcomes assessed through clinical instruments and/or digital phenotyping?

Relevant studies were retrieved and screened in full text by one author LB. After removing duplicates, LB, MP, and RA cross-checked the full texts against the inclusion and exclusion criteria. UH served as a supervisory reviewer. In an online meeting, final study selections were presented by LB and MP, who provided justification for inclusion decisions to UH.

### Inclusion Criteria

Studies were included if they met the following criteria:

1. Examined a digital intervention aimed at monitoring or improving depressive symptoms
2. Delivered via smartphone apps, wrist-worn devices, web-based platforms, offline software, or automated interactive voice response systems
3. Included adult participants (≥18 years) with depression, confirmed through validated clinical instruments (eg, PHQ-9)
4. Reported on outcomes related to monitoring and/or symptom improvement using digital phenotyping (passive sensor data and/or active user input)
5. Provided data on device use (eg, number of interventions delivered, duration of engagement)
6. Published in English or German, and accessible via open access or institutional access.

### Exclusion Criteria

Studies were excluded if they:

1. Focused on depression comorbid with other conditions (eg, cancer, substance use, pregnancy, or other mental health disorders)
2. Focused on depression in the context of a viral pandemic (eg, COVID-19)
3. Studies use or report on depression based on digital interventions aimed at supporting and improving physical activity [26].

### Analysis Approach

#### Data Extraction, Charting, and Synthesis 

A structured Microsoft Excel form was developed by the author LB to extract key information from each included study. Extracted variables included:

- Study characteristics (author, year, and country)
- Participant demographics and sample size
- Type of digital intervention and delivery platform
- Sensor types and data collection methods
- Depression assessment tools (baseline and follow-up)
- Frequency and duration of interventions
- Outcomes related to depression monitoring or prediction

Once data were initially charted, it was presented in an online meeting to co-author MP and cross-checked among author LB and co-author MP. For more detailed procedures for selection of the studies, extraction, and exclusion, please see Multimedia Appendix 1.

Due to heterogeneity in study design and reporting, a narrative synthesis approach was used. Data were charted and grouped into thematic categories of parameters (see Table 2), including physical activity and location, behavioral patterns, physiological signals, sleep indicators, and sociability and self-reported input.

**Table 2.** Overview of the retrieved studies in terms of data collection method, type of digital tool, depression measures, main findings, and predictive value for depression reported.

Study ID	Reference identifier	Sample size (depression)	Data type	Digital tool type	Depression measure	Main findings	Predictive value reported
1	[27]	313	Passive + Active	Smartphone app, wearable	PHQ-9^a^	Multiple behavioral and phone use features significantly correlated with PHQ-9	Yes
2	[28]	400	Active	Computer	PHQ-9	Behavioral test metrics significantly predicted depression	Yes
3	[29]	290	Passive	Fitbit	PHQ-9	Variations in heart rate and circadian rhythm linked to depression	Yes
4	[30]	92	Passive	Smartphone app	PHQ-9	User engagement is essential for data reliability	Unclear
5	[31]	81277	Active	Smartphone app	PHQ-9	Minimal average usage; thought diary and tests most used	No
6	[32]	934	Passive	Smartphone app	PHQ-2^b^	Weak correlation between keystroke timing and symptoms	No
7	[33]	65	Passive	Smartphone app	CES-D^c^	Subject-specific standardization improved forecasting	Yes
8	[13]	60	Passive + Active	Smartphone app, Oura Ring	DASS-21^d^	Best prediction from combined smartphone + wearable + mood	Yes
9	[34]	28	Active	Computer	PHQ-8^e^	Digital social rhythm therapy improved depression	Yes
10	[35]	614	Passive	Smartphone app, Fitbit	PHQ-8	Lower engagement correlated with higher depression	Unclear
11	[36]	1	Passive	Smartphone app, wearable	Not stated	Demonstrated potential of ambient light and usage data	Unclear
12	[37]	18	Passive	Video assessment	MADRS^f^, MINI^g^, CSSRS^h^	Facial and speech features correlated with MADRS	Yes
13	[38]	20	Passive + Active	Smartphone app, wearable	MADRS, HDRS^i^	Self-report had higher predictive correlation than passive sensors	Yes
14	[39]	623	Passive	Smartphone, Fitbit	PHQ-8	Sleep timing features correlated with depression severity	Yes
15	[20]	107	Active	Smartphone app	PHQ-9	Web-based support linked to symptom improvement	Yes
16	[40]	43	Active	Smartphone app	BDI^j^	Smartphone mood ratings correlated with standard assessments	Yes
17	[41]	48	Passive + Active	Smartphone app, wearable	PHQ-9	Higher engagement led to greater symptom improvement	Yes
18	[42]	136	Active	Smartphone app	BDI-II^k^	Significant treatment-related symptom changes	Yes
19	[43]	124	Active	Computer	DASS-21	Personalized feedback led to greater symptom reduction	Yes

a PHQ-9: Patient Health Questionnaire-9.

b PHQ-2: Patient Health Questionnaire-2.

c CES-D: Center for Epidemiological Studies Depression Scale.

d DASS-21: Depression Anxiety Stress Scale Short Form 21.

e PHQ-8: Patient Health Questionnaire-8.

f MADRS: Structured Interview Guide for the Montgomery-Åsberg Depression Rating Scale.

g MINI: Mini-International Neuropsychiatric Interview,

h CSSRS: Columbia Suicide Severity Rating Scale.

i HDRS: Hamilton Depression Rating Scale.

j BDI: Beck Depression Inventory.

k BDI-II: Beck Depression Inventory, Second Edition.

The frequency of each parameter category and its reported predictive value were also noted.

### Quality Appraisal

To assess the quality of the included studies, we used two tools, including (1) the Downs and Black checklist [44] used for randomized controlled trials and quantitative studies and (2) the Mixed Methods Appraisal Tool (MMAT) [45] used for mixed method and nonrandomized studies.

These complementary tools allowed a consistent evaluation of methodological rigor across diverse study designs. Detailed quality assessments are included in MultimediaAppendices 2^4^.

## Results

### Summary of Included Studies

**Figure 1.**
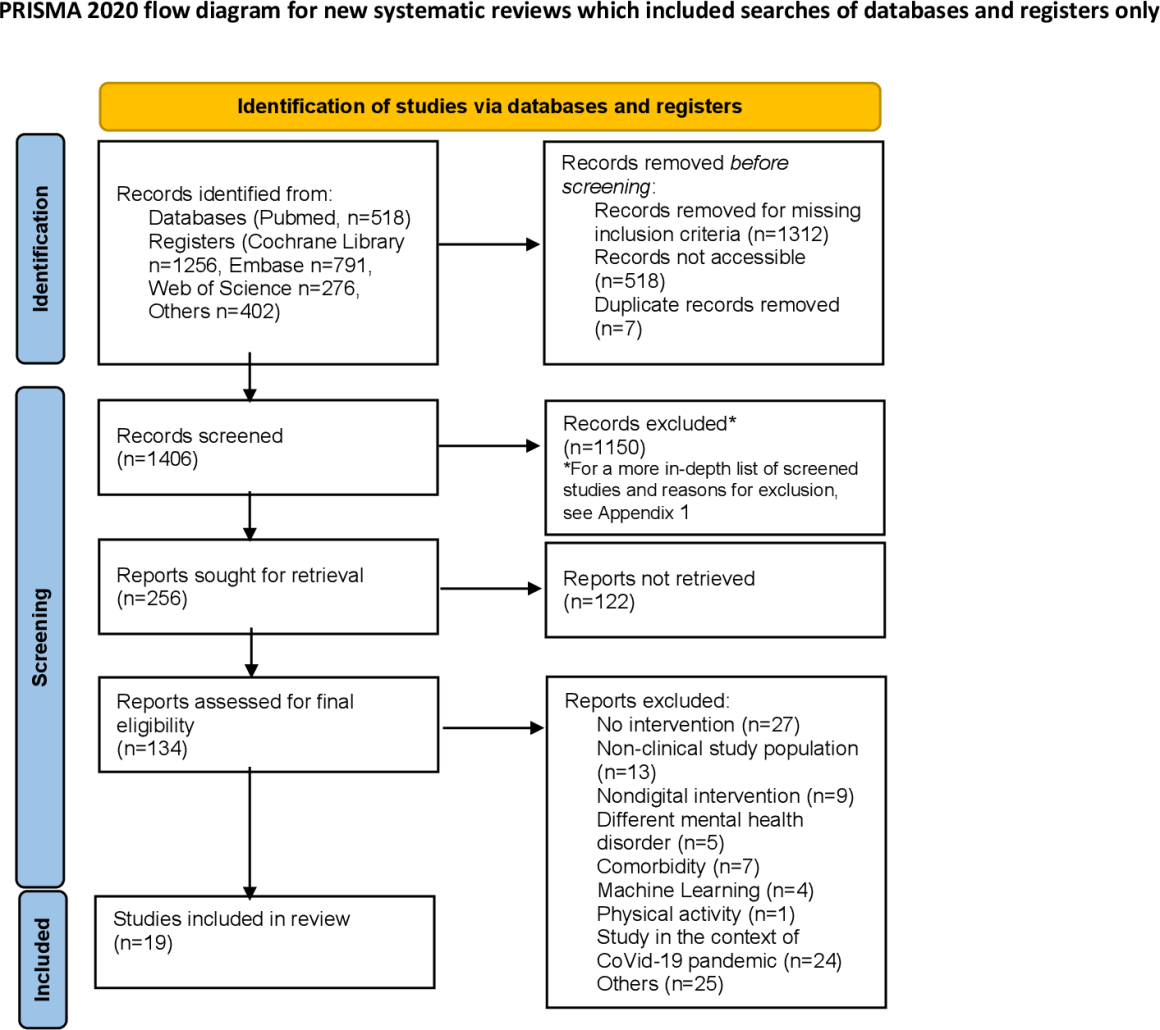
PRISMA-ScR (Preferred Reporting Items for Systematic Reviews and Meta-Analyses Extension for Scoping Reviews) flow diagram of the study selection process. Diagram adapted from PRISMA-ScR guidelines [24].

A total of 19 studies [13,20,27-43] with 85,193 participants across studies were included, covering a diverse range of digital intervention types and data collection methods. The study selection process is shown in Figure 1.

Most digital tools or platforms (n=15) used smartphone apps, either alone or in combination with wearable devices (eg, Fitbit [Fitbit, Inc], Oura Ring [Oura Health Oy]). Three studies (16%) used computer-based platforms, and one used a computer-based platform for video-based digital phenotyping. The included studies varied greatly in design and measurement approaches (see Table 3). The majority were observational studies (n=14), including cross-sectional, longitudinal cohort, retrospective, interformat validation, and feasibility designs, while 5 studies delivered a digital mental health intervention [20,34,41-43]. Three studies were randomized controlled trials [13,42,43], and 2 used a retrospective observational approach [30,39]. Several observational studies used repeated-measures designs to monitor depressive symptoms over time, although study duration and assessment frequency were heterogeneous. One study collected single-participant feasibility data [36], while another followed participants longitudinally for 43 weeks [35].

**Table 3.** Description of study design, subtype, number of studies included, and reference number.

Study design and subtype (if applicable)		N	Reference number
Randomized controlled trials (RCTs)		3	[34,42,43]
Nonrandomized interventional pilot studies			
Pilot intervention as well as feasibility with outcomes		2	[37,41]
Observational studies			
Cross-sectional		5	[28,29,31,32,38]
Prospective cohort and longitudinal cohort		5	[13,20,27,33,35]
Retrospective observational		2	[30,39]
Single-participant feasibility and case-style		1	[36]
Interformat validation		1	[40]

### Data Collection Approaches

Three data collection types were noted, including passive data collection, active input, self-reports, and multimodal approaches, which combined both passive and active inputs.

Passive data collection (eg, from GPS, accelerometer, and heart rate sensors) was used in 84% of studies (n=16).

Active input and self-reports (eg, mood ratings, diary entries) were used in 58% of studies (n=11).

Multimodal approaches combining both passive and active inputs showed the highest predictive potential and were used in 47 % of studies (n=9).

### Clinical Measures Used Across Studies

Validated tools used across studies included Patient Health Questionnaire versions 9, 8 and 2 (PHQ-9, PHQ-8, and PHQ-2), the Center for Epidemiologic Studies Depression Scale (CES-D), the Depression Anxiety and Stress Scale (DASS-21), the Beck Depression Inventory original and revised version (BDI-I and -II), the Montgomery-Åsbergg Depression Rating Scale (MADRS), the Hamilton Depression Rating Scale (HDRS), and the Mini-International Neuropsychiatric Interview (MINI), reflecting a broad clinical foundation across studies (see Table 2).

The PHQ-9 was the most used depression measure, used in 9 studies. The second most used depression measure included PHQ-8 (studies n=3) [34,35,39] while PHQ-2 was used in only one study [32]. Other often used measures were DASS (studies n=2) [13,43], MADRS (studies n=2) [37,38], MINI. (studies n=2) [37,38] and HDRS [38] used in only one study.

### Predictive Value

Of the 19 studies, 15 (79%) [13,20,27-29,33-35,37-43] reported predictive or diagnostic associations between digital parameters and depression outcomes. Predictive value was strongest in studies that:

1. Incorporated multisource data fusion [9,13,38]
2. Applied machine learning or modeling techniques for forecasting [28,33].

### Sensor Types and Platforms Breakdown

Across the reviewed studies, most digital interventions assessed or observed relied on smartphones as the primary platform, often enhanced with wearable devices (eg, Fitbit, Oura Ring) for additional collection of physiological data.

The most frequently used sensors included accelerometers, gyroscopes, GPS, light sensors, and microphone sensors.

Wearables primarily contributed heart rate and sleep cycle data, while smartphones captured mobility, screen interaction, ambient context, and social communication metrics.

Only a few studies [28,37] used computer-based platforms or video-based assessments, which focused on cognitive tasks, facial expressions, and speech expression as digital biomarkers.

Across retrieved studies the most common device used for digital data collection was a smartphone (n=13), while wearable devices such as sensors and other wrist-worn devices (eg, Fitbit) were used in 8 studies. A detailed list of devices with the main findings reported can be seen in Table 2.

### Key Findings

**Figure 2.**
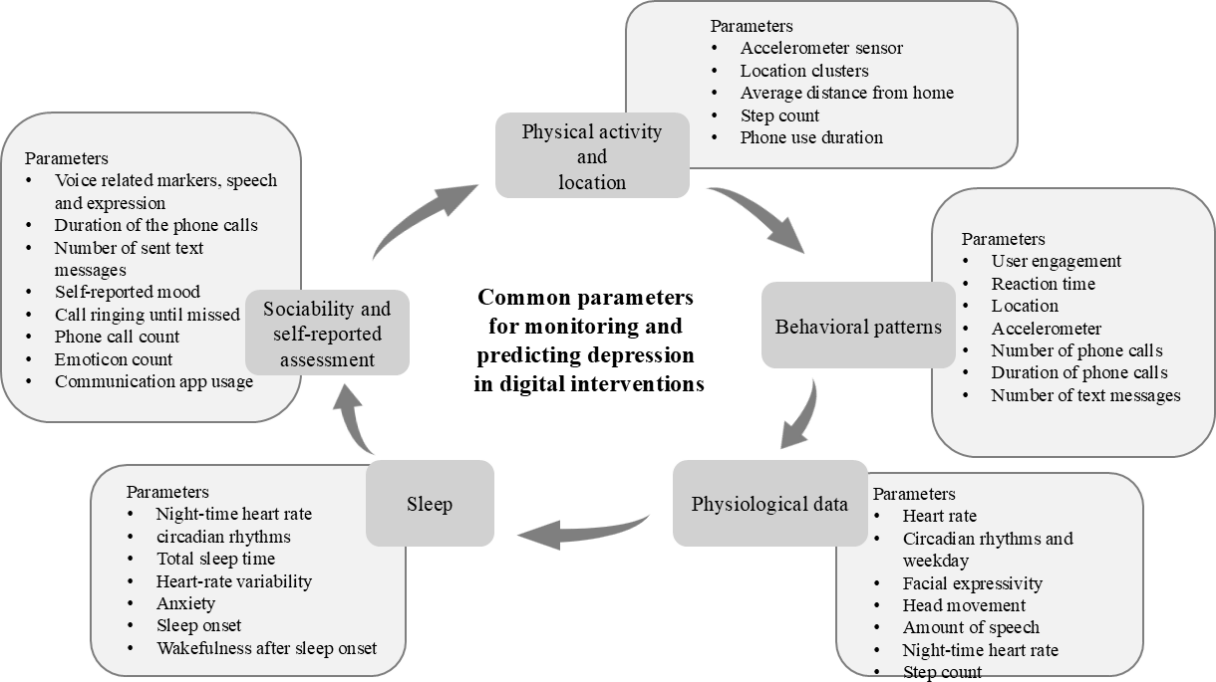
Common domains and parameters within domains used for digital phenotyping in depression monitoring.

The key findings were grouped into five major categories of passively collected parameters: (1) physical activity and location, (2) behavioral patterns, (3) physiological data*,* (4) sleep, and (5) sociability and self-reported assessments (see Table 2 and Figure 2). Table 2 summarizes the most commonly used passively collected parameters in digital interventions for depression. Sleep-related parameters such as total sleep time, sleep onset, and heart rate during sleep were particularly prevalent, often inferred through sensor fusion.

Similarly, physical activity metrics like step count and accelerometer data, along with location tracking (eg, distance from home), were commonly used to assess daily functioning and routine stability.

Sociability parameters, including phone call frequency, text message frequency, and app usage, were often used to approximate social withdrawal.

Behavioral data like reaction time or user engagement further contributed to characterizing depressive symptomatology. Figure 2 illustrates how these domains interconnect and highlights their contribution to monitoring and predicting depression.

This multidomain structure, used in 47% of studies, supports a more concrete understanding of symptom trajectories and lays a foundation for developing more reliable personalized digital interventions that incorporate digital parameters.

1. Physical Activity and location parameters—such as step count, phone use duration, and location-based data—were reported in 8 studies and captured through various digital interventions (see Table 4).
2. Behavioral patterns included parameters like user engagement, often assessed via response time to survey notifications. These were also reported in 8 studies.
3. Physiological data encompassed diverse indicators such as heart rate, circadian rhythms, and facial expressivity. These were observed in 8 studies and often overlapped conceptually with parameters categorized under “Sleep”, such as total sleep time, sleep onset, and nighttime heart rate. Sleep-related parameters were collected in 7 studies.
4. Sociability and self-reported assessments involved features inferred from communication behaviors, including the number and duration of phone calls, text message counts, and emoticon use. These were reported in 3 studies. Self-reported mood or symptom assessments, often gathered through in-app or EMA-style prompts, were present in nearly all included studies and frequently used alongside sensor data.

**Table 4.** Categories included in the monitoring and assessment of depression per study.

Study ID	Sleep	Physical activity	Sociability	Location	Physiological	Other	Total parameters measured
1	Yes	Yes	Yes	No	Yes	No	34
2	No	No	No	No	No	No	—^a^
3	Yes	Yes	Yes	No	Yes	No	126
4	No	Yes	No	Yes	No	No	—
5	No	No	Yes	Yes	No	No	—
6	Yes	No	No	No	No	Yes	54
7	Yes	No	No	Yes	No	Yes	19
8	Yes	Yes	No	Yes	Yes	Yes	—
9	No	No	No	No	No	Yes	—
10	No	No	No	No	No	No	—
11	No	Yes	No	Yes	No	Yes	5
12	No	Yes	No	No	Yes	Yes	—
13	No	No	No	No	No	No	—
14	Yes	No	No	Yes	No	Yes	21
15	No	No	No	No	No	Yes	—
16	Yes	Yes	No	Yes	No	Yes	—
17	No	Yes	No	Yes	Yes	Yes	—
18	No	No	No	No	No	Yes	—
19	No	No	No	No	No	No	—

a Not applicable.

Across the studies, the most robust prediction of depressive symptoms emerged from models that combined smartphone and wearable sensor data with self-reported assessments. Although there was considerable heterogeneity in the parameters used, ranging from location variability and facial expressivity to sleep duration and keystroke timing, some overlap was noted. For instance, nighttime heart rate could be classified under either physiological data or sleep, depending on the study context. In such cases, we categorized the parameter according to how it was used in the original study.

### Elaboration of Key Findings

#### Physical Activity and Location

The majority of studies that gathered passive data continually also supplied data about patient’s locations. Additionally, these studies tracked patients’ physical activity via accelerometer sensor. Results regarding physical activity and location parameters were homogenous. One study described a negative association between variability of locations visited and symptoms of depression (*r*=−0.21) [13], showing that the more active the participant is (ie, the more locations visited), the lower the symptoms of depression appear to be, which can also infer social context, as more activity could also signify more social interactions. Similarly, another study described a negative correlation between higher MADRS scores and a lower average distance from home (*r*=−0.25) [38], supporting the argument that greater depression severity is associated with reduced physical activity.

#### Behavioral Patterns

Studies categorized in behavioral patterns predominantly focused on user engagement and behavior of the patient. One of the included studies revealed that lower user engagement results in higher depression scores [41]. Although, the engagement in this case was defined as the time it took the user to respond to survey notifications. The longer the time users took to respond, the less engaged they were and the more severe their depressive symptoms were shown to be. In line with this, another study noted a correlation between higher engagement and an increase in symptom improvement, described as a decrease in at least one of the depressive symptoms and/or a clinically significant improvement [41]. User engagement can be used to assess the severity of depressive symptoms, and it is also important for gathering data continually, especially for gathering relevant data such as location and accelerometer; hence, active user engagement was found to be essential [30] also in supporting the physical activity and location category.

Apart from engagement rates, other behavioral markers differed in relation to depressive symptoms as well. A prospective cohort study, included in this review, exploring factors associated with the onset and course of mental health conditions during adolescence reported a weak negative association between most of the 54 analyzed keystroke timing features and mental health symptoms [32]. Machine learning models using keystroke features alone were shown to be unable to predict mental health symptoms, while speech patterns and other associated voice-related markers, such as words per minute, were found to be negatively correlated (*r*=−0.09) with PHQ-9 [27] indicating that higher depression scores are associated with decreased speech and expression, which potentially has value for the (5) sociability and self-reported assessments category as well.

#### Physiological Data

Common parameters in this category were difficult to compare, as they vary in nature. Multiple physiological markers were shown to correlate with the severity of depressive symptoms across studies. For instance, one study reported significant increases in multiple digital markers, especially in facial expressivity such as fear and anger [37]. These markers were shown to decrease often in frequency, correlating to changes in the MADRS scores. Concretely, head movement and amount of speech correlated with changes in the MADRS scores [37], while other physiological parameters were associated (*r*=0.70) with greater severity of depressive symptom, such as greater variation of nighttime heart rate [29]. Based on the daily number of steps, depressive symptoms were associated (*r*=0.62) with lower regularity of weekday circadian rhythms [29]. In fact, this particular study also reported overall a limited ability of digital biomarkers to detect depression in the whole sample [29]. However, stronger correlations (*r*=0.8‐1.0) were described among pairs of variables in alpha power and gamma power spectra in electroencephalography with MADRS [38], indicating that some pairs of variables in electroencephalographic streams can be measured when depression scores are increasing as well, being potentially useful as a digital biomarker in the prediction of depression.

#### Sleep

Multiple studies reported sleep-related data, consisting of passively gathered data or sleep-related questionnaires such as the Pittsburgh Sleep Quality Index, the Sleep Hygiene Index, or the Epworth Sleepiness Scale. Results were comparatively homogenous. One study reported a significant positive association between the total sleep time and depression (*r*=0.15), also waking after the sleep onset and depression (*r*=0.20), as well as heart rate variability and anxiety (*r*=0.12) [13]. Similarly, another study described a negative correlation (*r*=−0.12) between reported sleep duration and PHQ-9 [27]. Eventually, a stronger correlation of sleep-onset time with PHQ-8 was found cross-sectionally, and a higher correlation (*r*=0.24) of wakefulness after sleep-onset with PHQ-8 was also observed longitudinally [39].

#### Sociability and Self-Reported Assessments

In this category, studies included parameters such as self-reported mood or parameters pertaining to social behavior and sociability (eg, phone calls, text messages, and other communication apps). Since this is a rather broad range, comparison is difficult. In accordance with earlier described results, one study reported a correlation of PHQ-9 with voice-related markers such as Voice Diary words per minute, Voice Diary sentiment, and Voice Diary duration (Mood Study App, Verily Life Sciences LLC). Markers such as call ringing until missed minutes (*r*=0.10) showed a correlation, and phone call count (*r*=−0.06) showed a negative correlation as well, as did emoticon count in text messages (*r*=−0.06), battery percentage (*r*=−0.7), and ambient audio level (*r*=0.10) [27].

Another study described a negative correlation of high MADRS scores with lower entropy of the usage time of communication apps and lower total count of communication app usage (*r*=−0.42), indicating a decreasing sociability as depression scores were increasing. A strong positive correlation (*r*=0.9) was noted between self-reported outcomes and total MADRS score [38]. User behavior was also analyzed in a different study that described a typical person using the mental health app 3 times a day for a total of 12 minutes over the course of 90 days. The most frequently used tools in this study app were the Thought Diary (MoodTools, Inquiry Health LLC) and the self-assessment tests [31]. Significant treatment and time interactions for the BDI, GAD-7, Recovery Assessment Scale (RAS), Rosenberg Self-Esteem Scale (RSES), and Sheehan Disability Scale (SDS) were described [42]. Another study reported that participation in Cognitive Behavior Immersion (CBI) sessions via app reduced symptoms of anxiety and depression in patients (*r*=−1.5) [20].

A large portion of findings within this category relies on behavior data and as such is closely related to the behavioral patterns category. However, apart from user behavior, social patterns such as social support are found to be influencing the severity of depressive symptoms and the prediction thereof. Web-based social support was found to predict the improvement of depressive symptoms [20] while social-rhythm-focused digital interventions were shown to benefit patients significantly. In fact, a more rapid rate of improvement according to the PHQ-8 was shown compared to nonpersonalized interventions [34]. Additionally, one study reported a greater decline in DASS-21 scores using personalized feedback derived from the experience sampling method (ESM) compared to using nonpersonalized feedback [43].

The majority of studies apply several categories with a range of parameters used. Commonly, at least 4 categories were used for monitoring, assessing, or tracking depression across digital tools of the studies included (see Table 4). The type of device used to deliver the support was not shown as a relevant factor in effectiveness, and high comparability between smartphone-based and nonsmartphone-based questionnaire scores was reported [42]. Nevertheless, it must be noted that categories are interdependent, and all studies used parameters existing across categories, focusing on multiple aspects.

## Discussion

### Principal Findings

By focusing on parameters rather than platforms or algorithms, this review responds directly to calls for greater comparability in digital phenotyping research. This scoping review identified five major categories of passively collected parameters commonly used in digital interventions for depression: (1) physical activity and location*,* (2) behavioral patterns, (3) physiological data, (4) sleep, and (5) sociability and self-reported assessments. Most studies used a combination of smartphone-based and wearable sensors alongside self-reports. The strongest predictive value for depression outcomes was observed in studies that integrated multiple data sources, especially when combined with machine learning or subject-specific modeling approaches. Despite promising findings, the field remains methodologically fragmented and lacks standardization in both sensor use and clinical integration.

Five overall categories distinguished in this review contain relevant parameters for monitoring, assessing, and potentially predicting depression by combining self-assessment and digital phenotyping in digital interventions. The existing findings point that the categories of (1) physical activity and location, (2) behavioral patterns, (3) physiological data, (4) sleep, and (5) sociability and self-reported assessments appear across literature as interdependent categories, used across studies to monitor and predict depression by using various passively gathered data from parameters such as accelerometer sensor, location clusters, average distance from home, step count, phone usage, number of phone calls, heart rate, sleep time, and voice-related markers, among others. Moreover, additional parameters retrieved also include 11 frequently used clinical and self-assessment measurements such as PHQ-9, PHQ-2, CES-D, DASS-21, PHQ-8, MINI, MADRS, CSSRS, HDRS, BDI, and BDI-II (see Table 2).

This synthesis aligns with the World Health Organization guideline recommendations [21] for strengthening universal health coverage, where the role of digital health interventions is addressed particularly in line with the insufficient quality parameters that can assure optimal health care support [21]. While they emphasize the need for improved quality parameters in digital health, our findings indicate that such parameters are arguably not missing but are rather scattered across literature without comparative analysis. It might be argued that current work is one of the early efforts in the field to collect or summarize such parameters for digital health interventions overall in a more structured manner.

Previous research has often examined digital phenotyping and depression in isolation [8,19,46] or focused on either digital interventions [20,34,47-49] or productive models for symptom progression [13,33] without comprehensively linking all aspects. This review aims to contribute to bridging that gap by grouping relevant parameters into more standard categories.

Retrieved parameters in this review can be used as a comprehensive guideline in determining relevant aspects to be implemented into digital health interventions for depression to combine optimal sensors and self-assessment-based tools for monitoring and predicting depression outcomes. It can be argued that the interdependence of distinguished categories provides a more solid understanding for developing optimal digital health intervention in terms of monitoring and self-management support for depression. Combining these categories by implementing described parameters can support more beneficial outcomes for the end user of digital self-management tools for depression.

Synthesis of result sections of the included studies indicated that predicting depressive symptoms by combining clinical assessment and digital phenotyping is a promising approach for further improvement of digital interventions’ reliability and efficiency for individuals living with depressive symptoms and major depressive disorder. In a study using a transfer learning approach, an improvement of 12% for one-day-ahead forecasting was noted [33]. Moreover, using the MADRS score as a baseline for depression severity, the highest correlation of prediction was reported with user self-reported assessment [33]. Among other such assessments were the PHQ-2 and a model consisting of the Bond and Lader lethargic-energetic visual analog scale and the Bond and Lader interested-bored visual analog scale plus the N-Back working memory task [38].

The scores obtained in a smartphone-based intervention correlated with the ratings of nonsmartphone-based HDRS. Concretely, the smartphone-based single-item self-rating of mood correlated negatively (*r*=−0.54) with the BDI sum of scores as well [42], indicating that the lower the perceived mood of the user was, the higher the depression score. Therefore, participant self-reported assessments can potentially be valuable in predicting depression severity when combined with digital phenotyping for higher accuracy and reliability. In line with that, the findings support prior work showing that self-reported assessments when combined with sensor data, can meaningfully predict depressive symptom severity [34,39,48].

Reflecting on the device delivering the intervention with a combined model of smartphone features, wearable features, and self-reported mood items is also noted in one of the retrieved studies’ approaches [13].

However, this review also reveals substantial variability and methodological inconsistencies across studies. Among the retrieved studies there was a notable lack of standardization in contrast with the World Health Organization recommendations guidelines [21] addressing the quality parameters. Sample sizes, study and intervention duration, sampling frequencies, and types of intervention differed considerably. This was particularly evident when efforts for comparison during the charting procedures were made, mirroring concerns in literature about standardization and reproducibility [22,30,46]. Missing data was frequent, and most studies did not report a follow-up or provide justification for demographic admissions, therefore leading to the inclusion of studies assessing depression severity with validated measures with a notably limited amount of measurement scales used across studies. Taking into consideration the chronic nature of depression, it must be noted that short-term observations, less than 6 months, might also not be sufficient to successfully record meaningful changes in symptom trajectories or assess the sustained effectiveness of digital interventions for depression.

Across the included studies, it is important to distinguish between studies that primarily examined associations and those that validated predictive models based on digital data. Only a subset of studies (6/19) developed or evaluated models to predict depression status or symptom severity from digital signals, including work on behavioral and clinical modeling, cognitive task-based digital biomarkers, wearable-derived screening models, and sensor-plus-EMA-based forecasting [13,27-29,33,39]. The remaining studies (13/19) were observational, feasibility, validation, or intervention trials. These studies primarily characterized associations between digital parameters and depressive symptoms, evaluated feasibility and engagement, validated self-report formats, or tested the clinical effects of digital interventions without undertaking formal predictive model validation [20,30-32,34-38,40-43]. This distinction underlines that much of the evidence base still addresses which digital features relate to depression, whereas a smaller subset of studies evaluates how well these features can support robust and generalizable prediction.

Considering the rapidly developing nature of digital interventions and digital phenotyping in the mental health field, multiple challenges in conducting this review were noted. While digital phenotyping has been increasingly used to monitor symptoms and to gather vast amounts of passive data, discovering studies that combine digital phenotyping with digitally delivered interventions proved challenging. Although many studies address digital tools or passive monitoring separately, only a small percentage integrate both, further showing the fragmentation within the field. Additionally, the narrow focus of this review on depression and depressive symptoms exclusively led to discarding similar studies focused on other mental illnesses, narrowing our evidence pool. Nevertheless, the assessment of studies in our review was rather challenging due to the enormous number of articles addressing either digital interventions and depression or digital phenotyping and depression but rarely combining all these areas of interest together. This observation further aligns with literature where similar limitations have been noted previously in an umbrella review of smartphone sensors for monitoring depression [10]. In this review, studies often focused on generalized categories rather than specific parameters, restricting reproducibility and cross-study comparison.

### Strengths and Limitations

This review provides a structured synthesis of passively gathered parameters used in digital interventions for depression, supplementing existing reviews that focus exclusively on either digital phenotyping [8,46] or digital interventions [1,20,34,47,49]. By incorporating both, this review offers a clearer framework for understanding how sensor-derived data and self-reported assessments can be integrated in monitoring depression.

Although many high-quality systematic reviews and meta-analyses on digital phenotyping or digital interventions have been published, there is rather limited systematic literature synthesis known to the authors of this review that includes and combines all relevant terms such as “digital intervention,” “depression,” and “digital phenotyping.”

The review allows a structured overview of all relevant categories and parameters used in digital interventions that address depression with clinical measures and digital phenotyping methodology. An important strength of this review is the categorization of parameters. It allows a comparison across 5 interdependent categories, supporting efforts in the mental health field to determine and assure reliability in parameter use for future research and intervention designs, as well as clinical use, aligning also with World Health Organization recommendations [21].

Nonetheless, several limitations of this review need to be acknowledged. First, the review’s narrow scope, focusing exclusively on studies that used both validated depression assessments and digital phenotyping, may have excluded relevant work on related conditions or interventions that did not incorporate passive data collection. While this decision was made to enhance clarity and focus, it inevitably limited the breadth of the review, as noted in other recent scoping and mixed methods reviews as well [13,45]. Second, although passive data gathering enables the collection of large volumes of data over relatively short periods, a few studies reported that only a small proportion of the collected data was directly relevant to their hypotheses, raising concerns about potential publication bias or overinterpretation of limited findings.

Included studies mostly described individual interventions in detail, although many did not report the individual parameters measured but rather focused on larger terms, which we define in our study as categories. Even though general categories across studies were comparable, the lack of information on the exact data gathered through digital phenotyping leads to limited reproducibility of these studies as well as limited comparability with similar studies, thereby raising questions about the implications of such parameters for future studies. Missing data handling was an issue encountered in numerous studies, varying from a lack of explanation regarding the drop-out rates over user-related problems or confounders to basic demographic information. Similar methodological weaknesses have been described as well in prior meta-analyses of mental health data for depression [8,30,39].

Third, technical variability also limited comparison. Concretely, retrieved studies used different sensor types and different software, which could possibly influence data reliability. Technological difficulties were reported in some studies, though their influence on the reliability of data was rarely discussed. In line with that, few studies discussed the influence of signals being generated due to reasons outside of illness context as potential confounders (eg, the influence of weather conditions on the amount of time spent at home limiting both physical and social activity). Previous work has highlighted the need to better account for such nonclinical sources of signal variability [10,38].

Finally, this review confronts challenges comparable to those of many trials researching technology or digital devices, including limited transferability of results due to the nature of study populations. The users that participated in the included studies were for the most part younger people who are commonly more familiar and comfortable with the technologies used, apart from a few studies, and had only a few issues using the interventions as intended. However, this might not be the case with an older population, thus indicating that results may not necessarily be representative, an issue that has also been raised in other studies [31,35].

Although this review identifies promising directions for standardizing and optimizing digital interventions for depression, it also underlines the need for improved methodological consistency, demographic inclusivity, and clearer reporting of sensor-derived parameters in the future.

### Conclusion

This scoping review offers an innovative contribution by systematically identifying and synthesizing common digital parameters used across heterogeneous digital tools for monitoring and predicting depression. In contrast to existing reviews that predominantly focus on specific sensing modalities, predictive algorithms, or the effectiveness of digital interventions, this review adopts a parameter-centric perspective, enabling comparison across observational, predictive, and interventional study designs. By mapping convergent parameters irrespective of device type or analytic approach, the review provides a clearer understanding of which digital features are consistently associated with depressive symptoms and outcomes. This synthesis advances the field by supporting standardization efforts, guiding future predictive model development, and reducing fragmentation in digital mental health research. In real-world settings, these findings can inform the design of interoperable digital tools, facilitate integration into clinical monitoring workflows, and support personalized, data-driven depression management beyond controlled research environments.

## Supplementary material

10.2196/70840 Multimedia Appendix 1 Reasons for exclusion of screened studies.

10.2196/70840 Multimedia Appendix 2 Downs and Black Instrument assessment performed for each retrieved paper.

10.2196/70840 Multimedia Appendix 3 Mixed Methods Appraisal Tool, version 2018. Assessment performed for each retrieved paper.

10.2196/70840 Multimedia Appendix 4 Detailed quality assessment parameters of included studies.

10.2196/70840 Checklist 1 PRISMA-ScR checklist.

## Abbreviations

BDI: Beck Depression Inventory
BDI-II: Beck Depression Inventory, Second Edition
CES-D: Center for Epidemiological Studies Depression Scale
CSSRS: Columbia Suicide Severity Rating Scale
DASS-21: Depression Anxiety Stress Scale Short Form 21
HDRS: Hamilton Depression Rating Scale
ICTRP: International Clinical Trials Registry Platform
MADRS: Montgomery-Åsberg Depression Rating Scale
MINI: Mini-International Neuropsychiatric Interview
MMAT: Mixed Methods Appraisal Tool
PHQ-2: Patient Health Questionnaire-2
PHQ-8: Patient Health Questionnaire-8
PHQ-9: Patient Health Questionnaire-9
PRISMA-ScR: Preferred Reporting Items for Systematic Reviews and Meta-Analyses Extension for Scoping Reviews
PROSPERO: International Prospective Register of Systematic Reviews
RAS: Recovery Assessment Scale
RSES: Rosenberg Self-Esteem Scale
SDS: Sheehan Disability Scale
WHO: World Health Organization
