# Supplementary material for: Distinguishing Common Digital Phenotyping and Self-Report Parameters for Monitoring and Predicting Depression: Scoping Review
Source: JMIR Mhealth Uhealth. 2026 Mar 2;14:e70840. doi: 10.2196/70840 (PMC12954677; doi:10.2196/70840)
Supplement: Multimedia Appendix 1 [file mhealth-v14-e70840-s001.docx]

Appendix 1

Reasons for exclusion of the screened studies

| **Excluded record number** | **Reason for exclusion** |
| --- | --- |
| **E1** | Review, no clearly stated digital intervention |
| **E2** | Non-clinical study population |
| **E3** | Non-clinical study population |
| **E4** | Focussed on the possibility of integrating an engineering perspective |
| **E5** | Usage of H protons magnetic resonance spectroscopy as measure |
| **E6** | Non-clinical study population |
| **E7** | Study protocol, no results available yet |
| **E8** | Results not accessible |
| **E9** | Deployment of a construct / user persona instead of real-life participants |
| **E10** | Monitoring of symptoms, no intervention |
| **E11** | Non-clinical study population |
| **E12** | Comorbidity / different mental health disorder |
| **E13** | No digital intervention |
| **E14** | Recruitment of caregivers |
| **E15** | Non-clinical study population |
| **E16** | Non-clinical study population |
| **E17** | Non-clinical study population |
| **E18** | Multiple comorbidities as well as additional mental health disorders |
| **E19** | Using machine learning to construct a new endophenotype class |
| **E20** | Monitoring, no intervention |
| **E21** | Additional mental health disorder, outside of timeframe |
| **E22** | Focussed on machine learning |
| **E23** | Monitoring, no intervention |
| **E24** | Acceptability, no intervention |
| **E25** | Non-clinical study population |
| **E26** | No digitally delivered intervention |
| **E27** | Outside of timeframe |
| **E28** | Monitoring, no intervention |
| **E29** | Non-clinical study population |
| **E30** | No screening for depressive symptoms, outside of timeframe |
| **E31** | Participants with self-reported chronic illness, not further specified or objectified |
| **E32** | Monitoring via machine learning, no intervention |
| **E33** | Comorbidity: Diabetes, outside of time-frame |
| **E34** | Focussed on the age differences |
| **E35** | Screening, no intervention |
| **E36** | Non-clinical study population |
| **E37** | No intervention |
| **E38** | Comorbidity / different mental health disorder: Insomnia |
| **E39** | No intervention |
| **E40** | Intervention not only digitally but additionally with antidepressants as well |
| **E41** | Non-clinical study population |
| **E42** | Multitude of different mental health disorders / comorbidities |
| **E43** | Monitoring, no intervention |
| **E44** | Monitoring, no intervention, focussed on the additional burden of the pandemic |
| **E45** | Monitoring, no intervention |
| **E46** | Monitoring, no intervention |
| **E47** | Study protocol, additionally no intervention described |
| **E48** | Monitoring, no intervention |
| **E49** | Monitoring, no digitally delivered intervention |
| **E50** | Comorbidities: Diabetes, long-term health conditions such as multiple sclerosis, dementia and others |
| **E51** | Different mental health disorder: Bipolar Disorder |
| **E52** | Comorbidity: Chronic Kidney Disease |
| **E53** | Intervention is not delivered digitally |
| **E54** | Machine Learning and monitoring, no intervention |
| **E55** | Translational research, outside of timeframe |
| **E56** | Case reports |
| **E57** | Description of the Design of the messaging tool |
| **E58** | Monitoring via machine learning, outside of timeframe |
| **E59** | Acceptability study with a singular intervention |
| **E60** | Out of timeframe |
| **E61** | Focussed solely on suicidal thoughts, not other depressive symptoms |
| **E62** | No results available |
| **E63** | Outside of timeframe |
| **E64** | Intervention supportive to in-person therapy |
| **E65** | Feasibility Study, no intervention |
| **E66** | Physical activity-based intervention |
| **E67** | Monitoring, no intervention |
| **E68** | Blended therapy tool |
| **E69** | Monitoring, no intervention |
| **E70** | Physical activity-based intervention |
| **E71** | Feasibility study, no intervention |
| **E72** | Non-clinical study population |
| **E73** | No digitally delivered intervention |
| **E74** | Focussed on the normalization of iCBT delivery |
| **E75** | Monitoring, no intervention |
| **E76** | Study focusses on depression in the context of the CoVid-19 pandemic |
| **E77** | No results available yet |
| **E78** | No results available yet |
| **E79** | Study protocol, not yet recruiting |
| **E80** | No results available yet |
| **E81** | No results available yet |
| **E82** | No results available yet |
| **E83** | No results available yet |
| **E84** | No results available yet |
| **E85** | Non-digital intervention |
| **E86** | Withdrawn due to difficulties with recruitment |
| **E87** | Non-digital intervention |
| **E88** | No results available |
| **E89** | No results available yet |
| **E90** | No results available |
| **E91** | Not yet recruiting |
| **E92** | No results available |
| **E93** | No results available yet |
| **E94** | Non-clinical study population |
| **E95** | Different mental health disorder (social anxiety disorder) |
| **E96** | Comorbidity: schizophrenia |
| **E97** | Systematic review, meta-analysis |
| **E98** | Non-depressed study population, comorbidity: chronic pelvic pain |
| **E99** | No results available yet |
| **E100** | No results available yet |
| **E101** | Study protocol, not yet recruiting |
| **E102** | No results available |
| **E103** | No results available yet |
| **E104** | Still ongoing |
| **E105** | No results available |
| **E106** | Depression in the context of the pandemic |
| **E107** | Non-clinical study population |
| **E108** | Out of timeframe |
| **E109** | Monitoring, no intervention |
| **E110** | Monitoring, no intervention |
| **E111** | Outside of timeframe |

**Note.** Study IDs correspond to excluded records identified during full-text screening. Full bibliographic details are available upon request.
