# Supplementary material for: Distinguishing Common Digital Phenotyping and Self-Report Parameters for Monitoring and Predicting Depression: Scoping Review
Source: JMIR Mhealth Uhealth. 2026 Mar 2;14:e70840. doi: 10.2196/70840 (PMC12954677; doi:10.2196/70840)
Supplement: Multimedia Appendix 3 [file mhealth-v14-e70840-s003.docx]

Appendix 3

**Mixed Methods Appraisal Tool, version 2018**

Retrieved from: Hong QN, Pluye P, Fàbrgues S, Bartlett G, Boardman F, Cargo M, Dagenais P, Gagnon M-P, Griffiths F, Nicolau B, O’Cathain A, Rousseau M-C, Vedel I. Mixed Methods Appraisal Tool (MMAT), version 2018. Registration of Copyright (#148552), Canadian Intellectual Property Office, Industry Canada

**Reference identifier 42, Study identifier 1**

| **Category of study design** | **Methodological quality criteria** | **Responses** | | |  |
| --- | --- | --- | --- | --- | --- |
|  |  | Yes | No | Can’t tell | Comments |
| Screening questions (for all types) | S1. Are there clear research questions? | X |  |  |  |
|  | S2. Do the collected data allow to address the research question? | X |  |  |  |
|  | Further appraisal may not be feasible or appropriate when the answer is „No“ or „Can’t tell“ to one or both screening questions. |  |  |  |  |
| 1. Qualitative | 1.1 Is the qualitative approach appropriate to answer the research question? |  |  |  |  |
|  | 1.2 Are the qualitative data collection methods adequate to address the research question? |  |  |  |  |
|  | 1.3 Are the findings adequately derived from the data? |  |  |  |  |
|  | 1.4 Is the interpretation of the results sufficiently substantiated by data? |  |  |  |  |
|  | 1.5 Is there coherence between qualitative data sources, collection, analysis and interpretation? |  |  |  |  |
| 1. Quantitative randomized controlled trials | 2.1 Is randomization appropriately performed? |  |  |  |  |
|  | 2.2 Are the groups comparable at baseline? |  |  |  |  |
|  | 2.3 Are there complete outcome data? |  |  |  |  |
|  | 2.4 Are outcome assessors blinded to the intervention provided? |  |  |  |  |
|  | 2.5 Did the participants adhere to the assigned intervention? |  |  |  |  |
| 1. Quantitative non-randomized | 3.1 Are the participants representative of the target population? | X |  |  |  |
|  | 3.2 Are measurements appropriate regarding both the outcome and intervention (or exposure)? | X |  |  |  |
|  | 3.3 Are there complete outcome data? | X |  |  |  |
|  | 3.4 Are the confounders accounted for in the design and analysis? | X |  |  |  |
|  | 3.5 During the study period, is the intervention administered (or exposure occurred) as intended? | X |  |  |  |
| 1. Quantitative descriptive | 4.1 Is the sampling strategy relevant to address the research question? |  |  |  |  |
|  | 4.2 Is the sample representative of the target population? |  |  |  |  |
|  | 4.3 Are the measurements appropriate? |  |  |  |  |
|  | 4.4 Is the risk of nonresponse bias low? |  |  |  |  |
|  | 4.5 Is the statistical analysis appropriate to answer the research question? |  |  |  |  |
| 1. Mixed Methods | 5.1 Is there an adequate rationale for using a mixed methods design to address the research question? |  |  |  |  |
|  | 5.2 Are the different components of the study effectively integrated to answer the research question? |  |  |  |  |
|  | 5.3 Are the outputs of the integration of qualitative and quantitative components adequately interpreted? |  |  |  |  |
|  | 5.4 Are divergencies and inconsistencies between quantitative and qualitative results adequately addressed? |  |  |  |  |
|  | 5.5 Do the different components of the study adhere to the quality criteria of each tradition of the methods involved? |  |  |  |  |

**Reference identifier 41, Study identifier 2**

| **Category of study design** | **Methodological quality criteria** | **Responses** | | |  |
| --- | --- | --- | --- | --- | --- |
|  |  | Yes | No | Can’t tell | Comments |
| Screening questions (for all types) | S1. Are there clear research questions? | X |  |  |  |
|  | S2. Do the collected data allow to address the research question? | X |  |  |  |
|  | Further appraisal may not be feasible or appropriate when the answer is „No“ or „Can’t tell“ to one or both screening questions. |  |  |  |  |
| 1. Qualitative | 1.1 Is the qualitative approach appropriate to answer the research question? |  |  |  |  |
|  | 1.2 Are the qualitative data collection methods adequate to address the research question? |  |  |  |  |
|  | 1.3 Are the findings adequately derived from the data? |  |  |  |  |
|  | 1.4 Is the interpretation of the results sufficiently substantiated by data? |  |  |  |  |
|  | 1.5 Is there coherence between qualitative data sources, collection, analysis and interpretation? |  |  |  |  |
| 1. Quantitative randomized controlled trials | 2.1 Is randomization appropriately performed? |  |  |  |  |
|  | 2.2 Are the groups comparable at baseline? |  |  |  |  |
|  | 2.3 Are there complete outcome data? |  |  |  |  |
|  | 2.4 Are outcome assessors blinded to the intervention provided? |  |  |  |  |
|  | 2.5 Did the participants adhere to the assigned intervention? |  |  |  |  |
| 1. Quantitative non-randomized | 3.1 Are the participants representative of the target population? | X |  |  |  |
|  | 3.2 Are measurements appropriate regarding both the outcome and intervention (or exposure)? | X |  |  | Multiple regression model with metrics for each task |
|  | 3.3 Are there complete outcome data? | X |  |  |  |
|  | 3.4 Are the confounders accounted for in the design and analysis? | X |  |  |  |
|  | 3.5 During the study period, is the intervention administered (or exposure occurred) as intended? | X |  |  |  |
| 1. Quantitative descriptive | 4.1 Is the sampling strategy relevant to address the research question? |  |  |  |  |
|  | 4.2 Is the sample representative of the target population? |  |  |  |  |
|  | 4.3 Are the measurements appropriate? |  |  |  |  |
|  | 4.4 Is the risk of nonresponse bias low? |  |  |  |  |
|  | 4.5 Is the statistical analysis appropriate to answer the research question? |  |  |  |  |
| 1. Mixed Methods | 5.1 Is there an adequate rationale for using a mixed methods design to address the research question? |  |  |  |  |
|  | 5.2 Are the different components of the study effectively integrated to answer the research question? |  |  |  |  |
|  | 5.3 Are the outputs of the integration of qualitative and quantitative components adequately interpreted? |  |  |  |  |
|  | 5.4 Are divergencies and inconsistencies between quantitative and qualitative results adequately addressed? |  |  |  |  |
|  | 5.5 Do the different components of the study adhere to the quality criteria of each tradition of the methods involved? |  |  |  |  |

**Reference identifier 43, Study identifier 3**

| **Category of study design** | **Methodological quality criteria** | **Responses** | | | | |  |
| --- | --- | --- | --- | --- | --- | --- | --- |
|  |  | Yes | No | | Can’t tell | | Comments |
| Screening questions (for all types) | S1. Are there clear research questions? | X | |  | |  |  |
|  | S2. Do the collected data allow to address the research question? | X | |  | |  |  |
|  | Further appraisal may not be feasible or appropriate when the answer is „No“ or „Can’t tell“ to one or both screening questions. |  | |  | |  |  |
| 1. Qualitative | 1.1 Is the qualitative approach appropriate to answer the research question? |  | |  | |  |  |
|  | 1.2 Are the qualitative data collection methods adequate to address the research question? |  | |  | |  |  |
|  | 1.3 Are the findings adequately derived from the data? |  | |  | |  |  |
|  | 1.4 Is the interpretation of the results sufficiently substantiated by data? |  | |  | |  |  |
|  | 1.5 Is there coherence between qualitative data sources, collection, analysis and interpretation? |  | |  | |  |  |
| 1. Quantitative randomized controlled trials | 2.1 Is randomization appropriately performed? |  | |  | |  |  |
|  | 2.2 Are the groups comparable at baseline? |  | |  | |  |  |
|  | 2.3 Are there complete outcome data? |  | |  | |  |  |
|  | 2.4 Are outcome assessors blinded to the intervention provided? |  | |  | |  |  |
|  | 2.5 Did the participants adhere to the assigned intervention? |  | |  | |  |  |
| 1. Quantitative non-randomized | 3.1 Are the participants representative of the target population? | X |  | |  | |  |
|  | 3.2 Are measurements appropriate regarding both the outcome and intervention (or exposure)? | X |  | |  | | Set of biomarkers remained the same, use of different cut-off scores for different subsamples |
|  | 3.3 Are there complete outcome data? | X |  | |  | |  |
|  | 3.4 Are the confounders accounted for in the design and analysis? | X |  | |  | |  |
|  | 3.5 During the study period, is the intervention administered (or exposure occurred) as intended? | X |  | |  | |  |
| 1. Quantitative descriptive | 4.1 Is the sampling strategy relevant to address the research question? |  |  | |  | |  |
|  | 4.2 Is the sample representative of the target population? |  |  | |  | |  |
|  | 4.3 Are the measurements appropriate? |  |  | |  | |  |
|  | 4.4 Is the risk of nonresponse bias low? |  |  | |  | |  |
|  | 4.5 Is the statistical analysis appropriate to answer the research question? |  |  | |  | |  |
| 1. Mixed Methods | 5.1 Is there an adequate rationale for using a mixed methods design to address the research question? |  |  | |  | |  |
|  | 5.2 Are the different components of the study effectively integrated to answer the research question? |  |  | |  | |  |
|  | 5.3 Are the outputs of the integration of qualitative and quantitative components adequately interpreted? |  |  | |  | |  |
|  | 5.4 Are divergencies and inconsistencies between quantitative and qualitative results adequately addressed? |  |  | |  | |  |
|  | 5.5 Do the different components of the study adhere to the quality criteria of each tradition of the methods involved? |  |  | |  | |  |

**Reference identifier 33, Study identifier 4**

| **Category of study design** | **Methodological quality criteria** | **Responses** | | |  |
| --- | --- | --- | --- | --- | --- |
|  |  | Yes | No | Can’t tell | Comments |
| Screening questions (for all types) | S1. Are there clear research questions? | X |  |  |  |
|  | S2. Do the collected data allow to address the research question? | X |  |  |  |
|  | Further appraisal may not be feasible or appropriate when the answer is „No“ or „Can’t tell“ to one or both screening questions. |  |  |  |  |
| 1. Qualitative | 1.1 Is the qualitative approach appropriate to answer the research question? |  |  |  |  |
|  | 1.2 Are the qualitative data collection methods adequate to address the research question? |  |  |  |  |
|  | 1.3 Are the findings adequately derived from the data? |  |  |  |  |
|  | 1.4 Is the interpretation of the results sufficiently substantiated by data? |  |  |  |  |
|  | 1.5 Is there coherence between qualitative data sources, collection, analysis and interpretation? |  |  |  |  |
| 1. Quantitative randomized controlled trials | 2.1 Is randomization appropriately performed? |  |  |  |  |
|  | 2.2 Are the groups comparable at baseline? |  |  |  |  |
|  | 2.3 Are there complete outcome data? |  |  |  |  |
|  | 2.4 Are outcome assessors blinded to the intervention provided? |  |  |  |  |
|  | 2.5 Did the participants adhere to the assigned intervention? |  |  |  |  |
| 1. Quantitative non-randomized | 3.1 Are the participants representative of the target population? |  |  |  |  |
|  | 3.2 Are measurements appropriate regarding both the outcome and intervention (or exposure)? |  |  |  |  |
|  | 3.3 Are there complete outcome data? |  |  |  |  |
|  | 3.4 Are the confounders accounted for in the design and analysis? |  |  |  |  |
|  | 3.5 During the study period, is the intervention administered (or exposure occurred) as intended? |  |  |  |  |
| 1. Quantitative descriptive | 4.1 Is the sampling strategy relevant to address the research question? | X |  |  |  |
|  | 4.2 Is the sample representative of the target population? | X |  |  | 3 large cohorts |
|  | 4.3 Are the measurements appropriate? | X |  |  |  |
|  | 4.4 Is the risk of nonresponse bias low? |  | X |  | High non-response rates |
|  | 4.5 Is the statistical analysis appropriate to answer the research question? | X |  |  |  |
| 1. Mixed Methods | 5.1 Is there an adequate rationale for using a mixed methods design to address the research question? |  |  |  |  |
|  | 5.2 Are the different components of the study effectively integrated to answer the research question? |  |  |  |  |
|  | 5.3 Are the outputs of the integration of qualitative and quantitative components adequately interpreted? |  |  |  |  |
|  | 5.4 Are divergencies and inconsistencies between quantitative and qualitative results adequately addressed? |  |  |  |  |
|  | 5.5 Do the different components of the study adhere to the quality criteria of each tradition of the methods involved? |  |  |  |  |

**Reference identifier 44, Study identifier 5**

| **Category of study design** | **Methodological quality criteria** | **Responses** | | |  |
| --- | --- | --- | --- | --- | --- |
|  |  | Yes | No | Can’t tell | Comments |
| Screening questions (for all types) | S1. Are there clear research questions? | X |  |  |  |
|  | S2. Do the collected data allow to address the research question? | X |  |  |  |
|  | Further appraisal may not be feasible or appropriate when the answer is „No“ or „Can’t tell“ to one or both screening questions. |  |  |  |  |
| 1. Qualitative | 1.1 Is the qualitative approach appropriate to answer the research question? |  |  |  |  |
|  | 1.2 Are the qualitative data collection methods adequate to address the research question? |  |  |  |  |
|  | 1.3 Are the findings adequately derived from the data? |  |  |  |  |
|  | 1.4 Is the interpretation of the results sufficiently substantiated by data? |  |  |  |  |
|  | 1.5 Is there coherence between qualitative data sources, collection, analysis and interpretation? |  |  |  |  |
| 1. Quantitative randomized controlled trials | 2.1 Is randomization appropriately performed? |  |  |  |  |
|  | 2.2 Are the groups comparable at baseline? |  |  |  |  |
|  | 2.3 Are there complete outcome data? |  |  |  |  |
|  | 2.4 Are outcome assessors blinded to the intervention provided? |  |  |  |  |
|  | 2.5 Did the participants adhere to the assigned intervention? |  |  |  |  |
| 1. Quantitative non-randomized | 3.1 Are the participants representative of the target population? |  |  | X | Deidentification and data aggregation don’t allow individual user data |
|  | 3.2 Are measurements appropriate regarding both the outcome and intervention (or exposure)? | X |  |  |  |
|  | 3.3 Are there complete outcome data? | X |  |  |  |
|  | 3.4 Are the confounders accounted for in the design and analysis? |  |  | X |  |
|  | 3.5 During the study period, is the intervention administered (or exposure occurred) as intended? | X |  |  | There was no set time-frame / minimum intervention |
| 1. Quantitative descriptive | 4.1 Is the sampling strategy relevant to address the research question? |  |  |  |  |
|  | 4.2 Is the sample representative of the target population? |  |  |  |  |
|  | 4.3 Are the measurements appropriate? |  |  |  |  |
|  | 4.4 Is the risk of nonresponse bias low? |  |  |  |  |
|  | 4.5 Is the statistical analysis appropriate to answer the research question? |  |  |  |  |
| 1. Mixed Methods | 5.1 Is there an adequate rationale for using a mixed methods design to address the research question? |  |  |  |  |
|  | 5.2 Are the different components of the study effectively integrated to answer the research question? |  |  |  |  |
|  | 5.3 Are the outputs of the integration of qualitative and quantitative components adequately interpreted? |  |  |  |  |
|  | 5.4 Are divergencies and inconsistencies between quantitative and qualitative results adequately addressed? |  |  |  |  |
|  | 5.5 Do the different components of the study adhere to the quality criteria of each tradition of the methods involved? |  |  |  |  |

**Reference identifier 37, Study identifier 6**

| **Category of study design** | **Methodological quality criteria** | **Responses** | | | | |  |
| --- | --- | --- | --- | --- | --- | --- | --- |
|  |  | Yes | No | | Can’t tell | | Comments |
| Screening questions (for all types) | S1. Are there clear research questions? | X | |  | |  |  |
|  | S2. Do the collected data allow to address the research question? | X | |  | |  |  |
|  | Further appraisal may not be feasible or appropriate when the answer is „No“ or „Can’t tell“ to one or both screening questions. |  | |  | |  |  |
| 1. Qualitative | 1.1 Is the qualitative approach appropriate to answer the research question? |  | |  | |  |  |
|  | 1.2 Are the qualitative data collection methods adequate to address the research question? |  | |  | |  |  |
|  | 1.3 Are the findings adequately derived from the data? |  | |  | |  |  |
|  | 1.4 Is the interpretation of the results sufficiently substantiated by data? |  | |  | |  |  |
|  | 1.5 Is there coherence between qualitative data sources, collection, analysis and interpretation? |  | |  | |  |  |
| 1. Quantitative randomized controlled trials | 2.1 Is randomization appropriately performed? |  | |  | |  |  |
|  | 2.2 Are the groups comparable at baseline? |  | |  | |  |  |
|  | 2.3 Are there complete outcome data? |  | |  | |  |  |
|  | 2.4 Are outcome assessors blinded to the intervention provided? |  | |  | |  |  |
|  | 2.5 Did the participants adhere to the assigned intervention? |  | |  | |  |  |
| 1. Quantitative non-randomized | 3.1 Are the participants representative of the target population? | X |  | |  | |  |
|  | 3.2 Are measurements appropriate regarding both the outcome and intervention (or exposure)? | X |  | |  | | Bivariate correlations, adjustement of false discovery rates of P values to q values |
|  | 3.3 Are there complete outcome data? | X |  | |  | |  |
|  | 3.4 Are the confounders accounted for in the design and analysis? | X |  | |  | |  |
|  | 3.5 During the study period, is the intervention administered (or exposure occurred) as intended? | X |  | |  | |  |
| 1. Quantitative descriptive | 4.1 Is the sampling strategy relevant to address the research question? |  |  | |  | |  |
|  | 4.2 Is the sample representative of the target population? |  |  | |  | |  |
|  | 4.3 Are the measurements appropriate? |  |  | |  | |  |
|  | 4.4 Is the risk of nonresponse bias low? |  |  | |  | |  |
|  | 4.5 Is the statistical analysis appropriate to answer the research question? |  |  | |  | |  |
| 1. Mixed Methods | 5.1 Is there an adequate rationale for using a mixed methods design to address the research question? |  |  | |  | |  |
|  | 5.2 Are the different components of the study effectively integrated to answer the research question? |  |  | |  | |  |
|  | 5.3 Are the outputs of the integration of qualitative and quantitative components adequately interpreted? |  |  | |  | |  |
|  | 5.4 Are divergencies and inconsistencies between quantitative and qualitative results adequately addressed? |  |  | |  | |  |
|  | 5.5 Do the different components of the study adhere to the quality criteria of each tradition of the methods involved? |  |  | |  | |  |

**Reference identifier 40, Study identifier 7**

| **Category of study design** | **Methodological quality criteria** | **Responses** | | |  |
| --- | --- | --- | --- | --- | --- |
|  |  | Yes | No | Can’t tell | Comments |
| Screening questions (for all types) | S1. Are there clear research questions? | X |  |  |  |
|  | S2. Do the collected data allow to address the research question? | X |  |  |  |
|  | Further appraisal may not be feasible or appropriate when the answer is „No“ or „Can’t tell“ to one or both screening questions. |  |  |  |  |
| 1. Qualitative | 1.1 Is the qualitative approach appropriate to answer the research question? |  |  |  |  |
|  | 1.2 Are the qualitative data collection methods adequate to address the research question? |  |  |  |  |
|  | 1.3 Are the findings adequately derived from the data? |  |  |  |  |
|  | 1.4 Is the interpretation of the results sufficiently substantiated by data? |  |  |  |  |
|  | 1.5 Is there coherence between qualitative data sources, collection, analysis and interpretation? |  |  |  |  |
| 1. Quantitative randomized controlled trials | 2.1 Is randomization appropriately performed? |  |  |  |  |
|  | 2.2 Are the groups comparable at baseline? |  |  |  |  |
|  | 2.3 Are there complete outcome data? |  |  |  |  |
|  | 2.4 Are outcome assessors blinded to the intervention provided? |  |  |  |  |
|  | 2.5 Did the participants adhere to the assigned intervention? |  |  |  |  |
| 1. Quantitative non-randomized | 3.1 Are the participants representative of the target population? | X |  |  |  |
|  | 3.2 Are measurements appropriate regarding both the outcome and intervention (or exposure)? | X |  |  |  |
|  | 3.3 Are there complete outcome data? | X |  |  |  |
|  | 3.4 Are the confounders accounted for in the design and analysis? | X |  |  | Cutoff time-frame |
|  | 3.5 During the study period, is the intervention administered (or exposure occurred) as intended? | X |  |  |  |
| 1. Quantitative descriptive | 4.1 Is the sampling strategy relevant to address the research question? |  |  |  |  |
|  | 4.2 Is the sample representative of the target population? |  |  |  |  |
|  | 4.3 Are the measurements appropriate? |  |  |  |  |
|  | 4.4 Is the risk of nonresponse bias low? |  |  |  |  |
|  | 4.5 Is the statistical analysis appropriate to answer the research question? |  |  |  |  |
| 1. Mixed Methods | 5.1 Is there an adequate rationale for using a mixed methods design to address the research question? |  |  |  |  |
|  | 5.2 Are the different components of the study effectively integrated to answer the research question? |  |  |  |  |
|  | 5.3 Are the outputs of the integration of qualitative and quantitative components adequately interpreted? |  |  |  |  |
|  | 5.4 Are divergencies and inconsistencies between quantitative and qualitative results adequately addressed? |  |  |  |  |
|  | 5.5 Do the different components of the study adhere to the quality criteria of each tradition of the methods involved? |  |  |  |  |

**Reference identifier 13, Study identifier 8**

| **Category of study design** | **Methodological quality criteria** | **Responses** | | |  |
| --- | --- | --- | --- | --- | --- |
|  |  | Yes | No | Can’t tell | Comments |
| Screening questions (for all types) | S1. Are there clear research questions? | X |  |  |  |
|  | S2. Do the collected data allow to address the research question? | X |  |  |  |
|  | Further appraisal may not be feasible or appropriate when the answer is „No“ or „Can’t tell“ to one or both screening questions. |  |  |  |  |
| 1. Qualitative | 1.1 Is the qualitative approach appropriate to answer the research question? |  |  |  |  |
|  | 1.2 Are the qualitative data collection methods adequate to address the research question? |  |  |  |  |
|  | 1.3 Are the findings adequately derived from the data? |  |  |  |  |
|  | 1.4 Is the interpretation of the results sufficiently substantiated by data? |  |  |  |  |
|  | 1.5 Is there coherence between qualitative data sources, collection, analysis and interpretation? |  |  |  |  |
| 1. Quantitative randomized controlled trials | 2.1 Is randomization appropriately performed? |  |  |  |  |
|  | 2.2 Are the groups comparable at baseline? |  |  |  |  |
|  | 2.3 Are there complete outcome data? |  |  |  |  |
|  | 2.4 Are outcome assessors blinded to the intervention provided? |  |  |  |  |
|  | 2.5 Did the participants adhere to the assigned intervention? |  |  |  |  |
| 1. Quantitative non-randomized | 3.1 Are the participants representative of the target population? | X |  |  |  |
|  | 3.2 Are measurements appropriate regarding both the outcome and intervention (or exposure)? | X |  |  |  |
|  | 3.3 Are there complete outcome data? | X |  |  |  |
|  | 3.4 Are the confounders accounted for in the design and analysis? |  |  | X |  |
|  | 3.5 During the study period, is the intervention administered (or exposure occurred) as intended? | X |  |  |  |
| 1. Quantitative descriptive | 4.1 Is the sampling strategy relevant to address the research question? |  |  |  |  |
|  | 4.2 Is the sample representative of the target population? |  |  |  |  |
|  | 4.3 Are the measurements appropriate? |  |  |  |  |
|  | 4.4 Is the risk of nonresponse bias low? |  |  |  |  |
|  | 4.5 Is the statistical analysis appropriate to answer the research question? |  |  |  |  |
| 1. Mixed Methods | 5.1 Is there an adequate rationale for using a mixed methods design to address the research question? |  |  |  |  |
|  | 5.2 Are the different components of the study effectively integrated to answer the research question? |  |  |  |  |
|  | 5.3 Are the outputs of the integration of qualitative and quantitative components adequately interpreted? |  |  |  |  |
|  | 5.4 Are divergencies and inconsistencies between quantitative and qualitative results adequately addressed? |  |  |  |  |
|  | 5.5 Do the different components of the study adhere to the quality criteria of each tradition of the methods involved? |  |  |  |  |

**Reference identifier 30, Study identifier 9**

| **Category of study design** | **Methodological quality criteria** | **Responses** | | |  |
| --- | --- | --- | --- | --- | --- |
|  |  | Yes | No | Can’t tell | Comments |
| Screening questions (for all types) | S1. Are there clear research questions? | X |  |  |  |
|  | S2. Do the collected data allow to address the research question? | X |  |  |  |
|  | Further appraisal may not be feasible or appropriate when the answer is „No“ or „Can’t tell“ to one or both screening questions. |  |  |  |  |
| 1. Qualitative | 1.1 Is the qualitative approach appropriate to answer the research question? |  |  |  |  |
|  | 1.2 Are the qualitative data collection methods adequate to address the research question? |  |  |  |  |
|  | 1.3 Are the findings adequately derived from the data? |  |  |  |  |
|  | 1.4 Is the interpretation of the results sufficiently substantiated by data? |  |  |  |  |
|  | 1.5 Is there coherence between qualitative data sources, collection, analysis and interpretation? |  |  |  |  |
| 1. Quantitative randomized controlled trials | 2.1 Is randomization appropriately performed? | X |  |  |  |
|  | 2.2 Are the groups comparable at baseline? | X |  |  |  |
|  | 2.3 Are there complete outcome data? | X |  |  |  |
|  | 2.4 Are outcome assessors blinded to the intervention provided? |  | X |  |  |
|  | 2.5 Did the participants adhere to the assigned intervention? | X |  |  |  |
| 1. Quantitative non-randomized | 3.1 Are the participants representative of the target population? |  |  |  |  |
|  | 3.2 Are measurements appropriate regarding both the outcome and intervention (or exposure)? |  |  |  |  |
|  | 3.3 Are there complete outcome data? |  |  |  |  |
|  | 3.4 Are the confounders accounted for in the design and analysis? |  |  |  |  |
|  | 3.5 During the study period, is the intervention administered (or exposure occurred) as intended? |  |  |  |  |
| 1. Quantitative descriptive | 4.1 Is the sampling strategy relevant to address the research question? |  |  |  |  |
|  | 4.2 Is the sample representative of the target population? |  |  |  |  |
|  | 4.3 Are the measurements appropriate? |  |  |  |  |
|  | 4.4 Is the risk of nonresponse bias low? |  |  |  |  |
|  | 4.5 Is the statistical analysis appropriate to answer the research question? |  |  |  |  |
| 1. Mixed Methods | 5.1 Is there an adequate rationale for using a mixed methods design to address the research question? |  |  |  |  |
|  | 5.2 Are the different components of the study effectively integrated to answer the research question? |  |  |  |  |
|  | 5.3 Are the outputs of the integration of qualitative and quantitative components adequately interpreted? |  |  |  |  |
|  | 5.4 Are divergencies and inconsistencies between quantitative and qualitative results adequately addressed? |  |  |  |  |
|  | 5.5 Do the different components of the study adhere to the quality criteria of each tradition of the methods involved? |  |  |  |  |

**Reference identifier 36, Study identifier 10**

| **Category of study design** | **Methodological quality criteria** | **Responses** | | |  |
| --- | --- | --- | --- | --- | --- |
|  |  | Yes | No | Can’t tell | Comments |
| Screening questions (for all types) | S1. Are there clear research questions? | X |  |  |  |
|  | S2. Do the collected data allow to address the research question? | X |  |  |  |
|  | Further appraisal may not be feasible or appropriate when the answer is „No“ or „Can’t tell“ to one or both screening questions. |  |  |  |  |
| 1. Qualitative | 1.1 Is the qualitative approach appropriate to answer the research question? |  |  |  |  |
|  | 1.2 Are the qualitative data collection methods adequate to address the research question? |  |  |  |  |
|  | 1.3 Are the findings adequately derived from the data? |  |  |  |  |
|  | 1.4 Is the interpretation of the results sufficiently substantiated by data? |  |  |  |  |
|  | 1.5 Is there coherence between qualitative data sources, collection, analysis and interpretation? |  |  |  |  |
| 1. Quantitative randomized controlled trials | 2.1 Is randomization appropriately performed? |  |  |  |  |
|  | 2.2 Are the groups comparable at baseline? |  |  |  |  |
|  | 2.3 Are there complete outcome data? |  |  |  |  |
|  | 2.4 Are outcome assessors blinded to the intervention provided? |  |  |  |  |
|  | 2.5 Did the participants adhere to the assigned intervention? |  |  |  |  |
| 1. Quantitative non-randomized | 3.1 Are the participants representative of the target population? | X |  |  |  |
|  | 3.2 Are measurements appropriate regarding both the outcome and intervention (or exposure)? | X |  |  |  |
|  | 3.3 Are there complete outcome data? | X |  |  |  |
|  | 3.4 Are the confounders accounted for in the design and analysis? | X |  |  |  |
|  | 3.5 During the study period, is the intervention administered (or exposure occurred) as intended? | X |  |  | Minimum time-frame was met, no maximum defined |
| 1. Quantitative descriptive | 4.1 Is the sampling strategy relevant to address the research question? |  |  |  |  |
|  | 4.2 Is the sample representative of the target population? |  |  |  |  |
|  | 4.3 Are the measurements appropriate? |  |  |  |  |
|  | 4.4 Is the risk of nonresponse bias low? |  |  |  |  |
|  | 4.5 Is the statistical analysis appropriate to answer the research question? |  |  |  |  |
| 1. Mixed Methods | 5.1 Is there an adequate rationale for using a mixed methods design to address the research question? |  |  |  |  |
|  | 5.2 Are the different components of the study effectively integrated to answer the research question? |  |  |  |  |
|  | 5.3 Are the outputs of the integration of qualitative and quantitative components adequately interpreted? |  |  |  |  |
|  | 5.4 Are divergencies and inconsistencies between quantitative and qualitative results adequately addressed? |  |  |  |  |
|  | 5.5 Do the different components of the study adhere to the quality criteria of each tradition of the methods involved? |  |  |  |  |

**Reference identifier 35, Study identifier 11**

| **Category of study design** | **Methodological quality criteria** | **Responses** | | |  |
| --- | --- | --- | --- | --- | --- |
|  |  | Yes | No | Can’t tell | Comments |
| Screening questions (for all types) | S1. Are there clear research questions? | X |  |  |  |
|  | S2. Do the collected data allow to address the research question? | X |  |  |  |
|  | Further appraisal may not be feasible or appropriate when the answer is „No“ or „Can’t tell“ to one or both screening questions. |  |  |  |  |
| 1. Qualitative | 1.1 Is the qualitative approach appropriate to answer the research question? |  |  |  |  |
|  | 1.2 Are the qualitative data collection methods adequate to address the research question? |  |  |  |  |
|  | 1.3 Are the findings adequately derived from the data? |  |  |  |  |
|  | 1.4 Is the interpretation of the results sufficiently substantiated by data? |  |  |  |  |
|  | 1.5 Is there coherence between qualitative data sources, collection, analysis and interpretation? |  |  |  |  |
| 1. Quantitative randomized controlled trials | 2.1 Is randomization appropriately performed? |  |  |  |  |
|  | 2.2 Are the groups comparable at baseline? |  |  |  |  |
|  | 2.3 Are there complete outcome data? |  |  |  |  |
|  | 2.4 Are outcome assessors blinded to the intervention provided? |  |  |  |  |
|  | 2.5 Did the participants adhere to the assigned intervention? |  |  |  |  |
| 1. Quantitative non-randomized | 3.1 Are the participants representative of the target population? |  |  |  |  |
|  | 3.2 Are measurements appropriate regarding both the outcome and intervention (or exposure)? |  |  |  |  |
|  | 3.3 Are there complete outcome data? |  |  |  |  |
|  | 3.4 Are the confounders accounted for in the design and analysis? |  |  |  |  |
|  | 3.5 During the study period, is the intervention administered (or exposure occurred) as intended? |  |  |  |  |
| 1. Quantitative descriptive | 4.1 Is the sampling strategy relevant to address the research question? | X |  |  |  |
|  | 4.2 Is the sample representative of the target population? |  |  | X | No details were given |
|  | 4.3 Are the measurements appropriate? | X |  |  |  |
|  | 4.4 Is the risk of nonresponse bias low? | X |  |  | But the sample size was n = 1 |
|  | 4.5 Is the statistical analysis appropriate to answer the research question? | X |  |  |  |
| 1. Mixed Methods | 5.1 Is there an adequate rationale for using a mixed methods design to address the research question? |  |  |  |  |
|  | 5.2 Are the different components of the study effectively integrated to answer the research question? |  |  |  |  |
|  | 5.3 Are the outputs of the integration of qualitative and quantitative components adequately interpreted? |  |  |  |  |
|  | 5.4 Are divergencies and inconsistencies between quantitative and qualitative results adequately addressed? |  |  |  |  |
|  | 5.5 Do the different components of the study adhere to the quality criteria of each tradition of the methods involved? |  |  |  |  |

**Reference identifier 38, Study identifier 12**

| **Category of study design** | **Methodological quality criteria** | **Responses** | | |  |
| --- | --- | --- | --- | --- | --- |
|  |  | Yes | No | Can’t tell | Comments |
| Screening questions (for all types) | S1. Are there clear research questions? | X |  |  |  |
|  | S2. Do the collected data allow to address the research question? | X |  |  |  |
|  | Further appraisal may not be feasible or appropriate when the answer is „No“ or „Can’t tell“ to one or both screening questions. |  |  |  |  |
| 1. Qualitative | 1.1 Is the qualitative approach appropriate to answer the research question? |  |  |  |  |
|  | 1.2 Are the qualitative data collection methods adequate to address the research question? |  |  |  |  |
|  | 1.3 Are the findings adequately derived from the data? |  |  |  |  |
|  | 1.4 Is the interpretation of the results sufficiently substantiated by data? |  |  |  |  |
|  | 1.5 Is there coherence between qualitative data sources, collection, analysis and interpretation? |  |  |  |  |
| 1. Quantitative randomized controlled trials | 2.1 Is randomization appropriately performed? |  |  |  |  |
|  | 2.2 Are the groups comparable at baseline? |  |  |  |  |
|  | 2.3 Are there complete outcome data? |  |  |  |  |
|  | 2.4 Are outcome assessors blinded to the intervention provided? |  |  |  |  |
|  | 2.5 Did the participants adhere to the assigned intervention? |  |  |  |  |
| 1. Quantitative non-randomized | 3.1 Are the participants representative of the target population? | X |  |  |  |
|  | 3.2 Are measurements appropriate regarding both the outcome and intervention (or exposure)? | X |  |  |  |
|  | 3.3 Are there complete outcome data? | X |  |  |  |
|  | 3.4 Are the confounders accounted for in the design and analysis? |  |  | X |  |
|  | 3.5 During the study period, is the intervention administered (or exposure occurred) as intended? | X |  |  |  |
| 1. Quantitative descriptive | 4.1 Is the sampling strategy relevant to address the research question? |  |  |  |  |
|  | 4.2 Is the sample representative of the target population? |  |  |  |  |
|  | 4.3 Are the measurements appropriate? |  |  |  |  |
|  | 4.4 Is the risk of nonresponse bias low? |  |  |  |  |
|  | 4.5 Is the statistical analysis appropriate to answer the research question? |  |  |  |  |
| 1. Mixed Methods | 5.1 Is there an adequate rationale for using a mixed methods design to address the research question? |  |  |  |  |
|  | 5.2 Are the different components of the study effectively integrated to answer the research question? |  |  |  |  |
|  | 5.3 Are the outputs of the integration of qualitative and quantitative components adequately interpreted? |  |  |  |  |
|  | 5.4 Are divergencies and inconsistencies between quantitative and qualitative results adequately addressed? |  |  |  |  |
|  | 5.5 Do the different components of the study adhere to the quality criteria of each tradition of the methods involved? |  |  |  |  |

**Reference identifier 39, Study identifier 13**

| **Category of study design** | **Methodological quality criteria** | **Responses** | | |  |
| --- | --- | --- | --- | --- | --- |
|  |  | Yes | No | Can’t tell | Comments |
| Screening questions (for all types) | S1. Are there clear research questions? | X |  |  |  |
|  | S2. Do the collected data allow to address the research question? | X |  |  |  |
|  | Further appraisal may not be feasible or appropriate when the answer is „No“ or „Can’t tell“ to one or both screening questions. |  |  |  |  |
| 1. Qualitative | 1.1 Is the qualitative approach appropriate to answer the research question? |  |  |  |  |
|  | 1.2 Are the qualitative data collection methods adequate to address the research question? |  |  |  |  |
|  | 1.3 Are the findings adequately derived from the data? |  |  |  |  |
|  | 1.4 Is the interpretation of the results sufficiently substantiated by data? |  |  |  |  |
|  | 1.5 Is there coherence between qualitative data sources, collection, analysis and interpretation? |  |  |  |  |
| 1. Quantitative randomized controlled trials | 2.1 Is randomization appropriately performed? |  |  |  |  |
|  | 2.2 Are the groups comparable at baseline? |  |  |  |  |
|  | 2.3 Are there complete outcome data? |  |  |  |  |
|  | 2.4 Are outcome assessors blinded to the intervention provided? |  |  |  |  |
|  | 2.5 Did the participants adhere to the assigned intervention? |  |  |  |  |
| 1. Quantitative non-randomized | 3.1 Are the participants representative of the target population? | X |  |  |  |
|  | 3.2 Are measurements appropriate regarding both the outcome and intervention (or exposure)? | X |  |  |  |
|  | 3.3 Are there complete outcome data? | X |  |  |  |
|  | 3.4 Are the confounders accounted for in the design and analysis? | X |  |  |  |
|  | 3.5 During the study period, is the intervention administered (or exposure occurred) as intended? | X |  |  |  |
| 1. Quantitative descriptive | 4.1 Is the sampling strategy relevant to address the research question? |  |  |  |  |
|  | 4.2 Is the sample representative of the target population? |  |  |  |  |
|  | 4.3 Are the measurements appropriate? |  |  |  |  |
|  | 4.4 Is the risk of nonresponse bias low? |  |  |  |  |
|  | 4.5 Is the statistical analysis appropriate to answer the research question? |  |  |  |  |
| 1. Mixed Methods | 5.1 Is there an adequate rationale for using a mixed methods design to address the research question? |  |  |  |  |
|  | 5.2 Are the different components of the study effectively integrated to answer the research question? |  |  |  |  |
|  | 5.3 Are the outputs of the integration of qualitative and quantitative components adequately interpreted? |  |  |  |  |
|  | 5.4 Are divergencies and inconsistencies between quantitative and qualitative results adequately addressed? |  |  |  |  |
|  | 5.5 Do the different components of the study adhere to the quality criteria of each tradition of the methods involved? |  |  |  |  |

**References identifier 34, Study identifier 14**

| **Category of study design** | **Methodological quality criteria** | **Responses** | | |  |
| --- | --- | --- | --- | --- | --- |
|  |  | Yes | No | Can’t tell | Comments |
| Screening questions (for all types) | S1. Are there clear research questions? | X |  |  |  |
|  | S2. Do the collected data allow to address the research question? | X |  |  |  |
|  | Further appraisal may not be feasible or appropriate when the answer is „No“ or „Can’t tell“ to one or both screening questions. |  |  |  |  |
| 1. Qualitative | 1.1 Is the qualitative approach appropriate to answer the research question? |  |  |  |  |
|  | 1.2 Are the qualitative data collection methods adequate to address the research question? |  |  |  |  |
|  | 1.3 Are the findings adequately derived from the data? |  |  |  |  |
|  | 1.4 Is the interpretation of the results sufficiently substantiated by data? |  |  |  |  |
|  | 1.5 Is there coherence between qualitative data sources, collection, analysis and interpretation? |  |  |  |  |
| 1. Quantitative randomized controlled trials | 2.1 Is randomization appropriately performed? |  |  |  |  |
|  | 2.2 Are the groups comparable at baseline? |  |  |  |  |
|  | 2.3 Are there complete outcome data? |  |  |  |  |
|  | 2.4 Are outcome assessors blinded to the intervention provided? |  |  |  |  |
|  | 2.5 Did the participants adhere to the assigned intervention? |  |  |  |  |
| 1. Quantitative non-randomized | 3.1 Are the participants representative of the target population? | X |  |  |  |
|  | 3.2 Are measurements appropriate regarding both the outcome and intervention (or exposure)? | X |  |  | Gaussian mixture model |
|  | 3.3 Are there complete outcome data? | X |  |  |  |
|  | 3.4 Are the confounders accounted for in the design and analysis? | X |  |  |  |
|  | 3.5 During the study period, is the intervention administered (or exposure occurred) as intended? | X |  |  |  |
| 1. Quantitative descriptive | 4.1 Is the sampling strategy relevant to address the research question? |  |  |  |  |
|  | 4.2 Is the sample representative of the target population? |  |  |  |  |
|  | 4.3 Are the measurements appropriate? |  |  |  |  |
|  | 4.4 Is the risk of nonresponse bias low? |  |  |  |  |
|  | 4.5 Is the statistical analysis appropriate to answer the research question? |  |  |  |  |
| 1. Mixed Methods | 5.1 Is there an adequate rationale for using a mixed methods design to address the research question? |  |  |  |  |
|  | 5.2 Are the different components of the study effectively integrated to answer the research question? |  |  |  |  |
|  | 5.3 Are the outputs of the integration of qualitative and quantitative components adequately interpreted? |  |  |  |  |
|  | 5.4 Are divergencies and inconsistencies between quantitative and qualitative results adequately addressed? |  |  |  |  |
|  | 5.5 Do the different components of the study adhere to the quality criteria of each tradition of the methods involved? |  |  |  |  |

**References identifier 20, Study identifier 15**

| **Category of study design** | **Methodological quality criteria** | **Responses** | | |  |
| --- | --- | --- | --- | --- | --- |
|  |  | Yes | No | Can’t tell | Comments |
| Screening questions (for all types) | S1. Are there clear research questions? | X |  |  |  |
|  | S2. Do the collected data allow to address the research question? | X |  |  |  |
|  | Further appraisal may not be feasible or appropriate when the answer is „No“ or „Can’t tell“ to one or both screening questions. |  |  |  |  |
| 1. Qualitative | 1.1 Is the qualitative approach appropriate to answer the research question? |  |  |  |  |
|  | 1.2 Are the qualitative data collection methods adequate to address the research question? |  |  |  |  |
|  | 1.3 Are the findings adequately derived from the data? |  |  |  |  |
|  | 1.4 Is the interpretation of the results sufficiently substantiated by data? |  |  |  |  |
|  | 1.5 Is there coherence between qualitative data sources, collection, analysis and interpretation? |  |  |  |  |
| 1. Quantitative randomized controlled trials | 2.1 Is randomization appropriately performed? |  |  |  |  |
|  | 2.2 Are the groups comparable at baseline? |  |  |  |  |
|  | 2.3 Are there complete outcome data? |  |  |  |  |
|  | 2.4 Are outcome assessors blinded to the intervention provided? |  |  |  |  |
|  | 2.5 Did the participants adhere to the assigned intervention? |  |  |  |  |
| 1. Quantitative non-randomized | 3.1 Are the participants representative of the target population? | X |  |  |  |
|  | 3.2 Are measurements appropriate regarding both the outcome and intervention (or exposure)? | X |  |  |  |
|  | 3.3 Are there complete outcome data? | X |  |  |  |
|  | 3.4 Are the confounders accounted for in the design and analysis? |  |  | X |  |
|  | 3.5 During the study period, is the intervention administered (or exposure occurred) as intended? | X |  |  |  |
| 1. Quantitative descriptive | 4.1 Is the sampling strategy relevant to address the research question? |  |  |  |  |
|  | 4.2 Is the sample representative of the target population? |  |  |  |  |
|  | 4.3 Are the measurements appropriate? |  |  |  |  |
|  | 4.4 Is the risk of nonresponse bias low? |  |  |  |  |
|  | 4.5 Is the statistical analysis appropriate to answer the research question? |  |  |  |  |
| 1. Mixed Methods | 5.1 Is there an adequate rationale for using a mixed methods design to address the research question? |  |  |  |  |
|  | 5.2 Are the different components of the study effectively integrated to answer the research question? |  |  |  |  |
|  | 5.3 Are the outputs of the integration of qualitative and quantitative components adequately interpreted? |  |  |  |  |
|  | 5.4 Are divergencies and inconsistencies between quantitative and qualitative results adequately addressed? |  |  |  |  |
|  | 5.5 Do the different components of the study adhere to the quality criteria of each tradition of the methods involved? |  |  |  |  |

**References identifier 49, Study identifier 16**

| **Category of study design** | **Methodological quality criteria** | **Responses** | | |  |
| --- | --- | --- | --- | --- | --- |
|  |  | Yes | No | Can’t tell | Comments |
| Screening questions (for all types) | S1. Are there clear research questions? | X |  |  |  |
|  | S2. Do the collected data allow to address the research question? | X |  |  |  |
|  | Further appraisal may not be feasible or appropriate when the answer is „No“ or „Can’t tell“ to one or both screening questions. |  |  |  |  |
| 1. Qualitative | 1.1 Is the qualitative approach appropriate to answer the research question? |  |  |  |  |
|  | 1.2 Are the qualitative data collection methods adequate to address the research question? |  |  |  |  |
|  | 1.3 Are the findings adequately derived from the data? |  |  |  |  |
|  | 1.4 Is the interpretation of the results sufficiently substantiated by data? |  |  |  |  |
|  | 1.5 Is there coherence between qualitative data sources, collection, analysis and interpretation? |  |  |  |  |
| 1. Quantitative randomized controlled trials | 2.1 Is randomization appropriately performed? |  |  |  |  |
|  | 2.2 Are the groups comparable at baseline? |  |  |  |  |
|  | 2.3 Are there complete outcome data? |  |  |  |  |
|  | 2.4 Are outcome assessors blinded to the intervention provided? |  |  |  |  |
|  | 2.5 Did the participants adhere to the assigned intervention? |  |  |  |  |
| 1. Quantitative non-randomized | 3.1 Are the participants representative of the target population? | X |  |  |  |
|  | 3.2 Are measurements appropriate regarding both the outcome and intervention (or exposure)? | X |  |  |  |
|  | 3.3 Are there complete outcome data? | X |  |  |  |
|  | 3.4 Are the confounders accounted for in the design and analysis? | X |  |  |  |
|  | 3.5 During the study period, is the intervention administered (or exposure occurred) as intended? | X |  |  |  |
| 1. Quantitative descriptive | 4.1 Is the sampling strategy relevant to address the research question? |  |  |  |  |
|  | 4.2 Is the sample representative of the target population? |  |  |  |  |
|  | 4.3 Are the measurements appropriate? |  |  |  |  |
|  | 4.4 Is the risk of nonresponse bias low? |  |  |  |  |
|  | 4.5 Is the statistical analysis appropriate to answer the research question? |  |  |  |  |
| 1. Mixed Methods | 5.1 Is there an adequate rationale for using a mixed methods design to address the research question? |  |  |  |  |
|  | 5.2 Are the different components of the study effectively integrated to answer the research question? |  |  |  |  |
|  | 5.3 Are the outputs of the integration of qualitative and quantitative components adequately interpreted? |  |  |  |  |
|  | 5.4 Are divergencies and inconsistencies between quantitative and qualitative results adequately addressed? |  |  |  |  |
|  | 5.5 Do the different components of the study adhere to the quality criteria of each tradition of the methods involved? |  |  |  |  |

**References identifier 31, Study identifier 17**

| **Category of study design** | **Methodological quality criteria** | **Responses** | | |  |
| --- | --- | --- | --- | --- | --- |
|  |  | Yes | No | Can’t tell | Comments |
| Screening questions (for all types) | S1. Are there clear research questions? | X |  |  |  |
|  | S2. Do the collected data allow to address the research question? | X |  |  |  |
|  | Further appraisal may not be feasible or appropriate when the answer is „No“ or „Can’t tell“ to one or both screening questions. |  |  |  |  |
| 1. Qualitative | 1.1 Is the qualitative approach appropriate to answer the research question? |  |  |  |  |
|  | 1.2 Are the qualitative data collection methods adequate to address the research question? |  |  |  |  |
|  | 1.3 Are the findings adequately derived from the data? |  |  |  |  |
|  | 1.4 Is the interpretation of the results sufficiently substantiated by data? |  |  |  |  |
|  | 1.5 Is there coherence between qualitative data sources, collection, analysis and interpretation? |  |  |  |  |
| 1. Quantitative randomized controlled trials | 2.1 Is randomization appropriately performed? |  |  |  |  |
|  | 2.2 Are the groups comparable at baseline? |  |  |  |  |
|  | 2.3 Are there complete outcome data? |  |  |  |  |
|  | 2.4 Are outcome assessors blinded to the intervention provided? |  |  |  |  |
|  | 2.5 Did the participants adhere to the assigned intervention? |  |  |  |  |
| 1. Quantitative non-randomized | 3.1 Are the participants representative of the target population? | X |  |  |  |
|  | 3.2 Are measurements appropriate regarding both the outcome and intervention (or exposure)? | X |  |  |  |
|  | 3.3 Are there complete outcome data? |  | X |  | Retention rate 65% |
|  | 3.4 Are the confounders accounted for in the design and analysis? |  |  | X |  |
|  | 3.5 During the study period, is the intervention administered (or exposure occurred) as intended? | X |  |  |  |
| 1. Quantitative descriptive | 4.1 Is the sampling strategy relevant to address the research question? |  |  |  |  |
|  | 4.2 Is the sample representative of the target population? |  |  |  |  |
|  | 4.3 Are the measurements appropriate? |  |  |  |  |
|  | 4.4 Is the risk of nonresponse bias low? |  |  |  |  |
|  | 4.5 Is the statistical analysis appropriate to answer the research question? |  |  |  |  |
| 1. Mixed Methods | 5.1 Is there an adequate rationale for using a mixed methods design to address the research question? |  |  |  |  |
|  | 5.2 Are the different components of the study effectively integrated to answer the research question? |  |  |  |  |
|  | 5.3 Are the outputs of the integration of qualitative and quantitative components adequately interpreted? |  |  |  |  |
|  | 5.4 Are divergencies and inconsistencies between quantitative and qualitative results adequately addressed? |  |  |  |  |
|  | 5.5 Do the different components of the study adhere to the quality criteria of each tradition of the methods involved? |  |  |  |  |

**References identifier 32, Study identifier 18**

| **Category of study design** | **Methodological quality criteria** | **Responses** | | | | |  |
| --- | --- | --- | --- | --- | --- | --- | --- |
|  |  | Yes | No | | Can’t tell | | Comments |
| Screening questions (for all types) | S1. Are there clear research questions? | X | |  | |  |  |
|  | S2. Do the collected data allow to address the research question? | X | |  | |  |  |
|  | Further appraisal may not be feasible or appropriate when the answer is „No“ or „Can’t tell“ to one or both screening questions. |  | |  | |  |  |
| 1. Qualitative | 1.1 Is the qualitative approach appropriate to answer the research question? |  | |  | |  |  |
|  | 1.2 Are the qualitative data collection methods adequate to address the research question? |  | |  | |  |  |
|  | 1.3 Are the findings adequately derived from the data? |  | |  | |  |  |
|  | 1.4 Is the interpretation of the results sufficiently substantiated by data? |  | |  | |  |  |
|  | 1.5 Is there coherence between qualitative data sources, collection, analysis and interpretation? |  | |  | |  |  |
| 1. Quantitative randomized controlled trials | 2.1 Is randomization appropriately performed? | X | |  | |  |  |
|  | 2.2 Are the groups comparable at baseline? |  | |  | | X | Different mental health disorders, but not spereated in the beginning |
|  | 2.3 Are there complete outcome data? | X | |  | |  |  |
|  | 2.4 Are outcome assessors blinded to the intervention provided? |  | | X | |  |  |
|  | 2.5 Did the participants adhere to the assigned intervention? | X | |  | |  |  |
| 1. Quantitative non-randomized | 3.1 Are the participants representative of the target population? |  |  | |  | |  |
|  | 3.2 Are measurements appropriate regarding both the outcome and intervention (or exposure)? |  |  | |  | |  |
|  | 3.3 Are there complete outcome data? |  |  | |  | |  |
|  | 3.4 Are the confounders accounted for in the design and analysis? |  |  | |  | |  |
|  | 3.5 During the study period, is the intervention administered (or exposure occurred) as intended? |  |  | |  | |  |
| 1. Quantitative descriptive | 4.1 Is the sampling strategy relevant to address the research question? |  |  | |  | |  |
|  | 4.2 Is the sample representative of the target population? |  |  | |  | |  |
|  | 4.3 Are the measurements appropriate? |  |  | |  | |  |
|  | 4.4 Is the risk of nonresponse bias low? |  |  | |  | |  |
|  | 4.5 Is the statistical analysis appropriate to answer the research question? |  |  | |  | |  |
| 1. Mixed Methods | 5.1 Is there an adequate rationale for using a mixed methods design to address the research question? |  |  | |  | |  |
|  | 5.2 Are the different components of the study effectively integrated to answer the research question? |  |  | |  | |  |
|  | 5.3 Are the outputs of the integration of qualitative and quantitative components adequately interpreted? |  |  | |  | |  |
|  | 5.4 Are divergencies and inconsistencies between quantitative and qualitative results adequately addressed? |  |  | |  | |  |
|  | 5.5 Do the different components of the study adhere to the quality criteria of each tradition of the methods involved? |  |  | |  | |  |

**References identifier 29, Study identifier 19**

| **Category of study design** | **Methodological quality criteria** | **Responses** | | | | |  |
| --- | --- | --- | --- | --- | --- | --- | --- |
|  |  | Yes | No | | Can’t tell | | Comments |
| Screening questions (for all types) | S1. Are there clear research questions? | X | |  | |  |  |
|  | S2. Do the collected data allow to address the research question? | X | |  | |  |  |
|  | Further appraisal may not be feasible or appropriate when the answer is „No“ or „Can’t tell“ to one or both screening questions. |  | |  | |  |  |
| 1. Qualitative | 1.1 Is the qualitative approach appropriate to answer the research question? |  | |  | |  |  |
|  | 1.2 Are the qualitative data collection methods adequate to address the research question? |  | |  | |  |  |
|  | 1.3 Are the findings adequately derived from the data? |  | |  | |  |  |
|  | 1.4 Is the interpretation of the results sufficiently substantiated by data? |  | |  | |  |  |
|  | 1.5 Is there coherence between qualitative data sources, collection, analysis and interpretation? |  | |  | |  |  |
| 1. Quantitative randomized controlled trials | 2.1 Is randomization appropriately performed? | X | |  | |  |  |
|  | 2.2 Are the groups comparable at baseline? | X | |  | |  |  |
|  | 2.3 Are there complete outcome data? | X | |  | |  |  |
|  | 2.4 Are outcome assessors blinded to the intervention provided? |  | |  | | X |  |
|  | 2.5 Did the participants adhere to the assigned intervention? | X | |  | |  |  |
| 1. Quantitative non-randomized | 3.1 Are the participants representative of the target population? |  |  | |  | |  |
|  | 3.2 Are measurements appropriate regarding both the outcome and intervention (or exposure)? |  |  | |  | |  |
|  | 3.3 Are there complete outcome data? |  |  | |  | |  |
|  | 3.4 Are the confounders accounted for in the design and analysis? |  |  | |  | |  |
|  | 3.5 During the study period, is the intervention administered (or exposure occurred) as intended? |  |  | |  | |  |
| 1. Quantitative descriptive | 4.1 Is the sampling strategy relevant to address the research question? |  |  | |  | |  |
|  | 4.2 Is the sample representative of the target population? |  |  | |  | |  |
|  | 4.3 Are the measurements appropriate? |  |  | |  | |  |
|  | 4.4 Is the risk of nonresponse bias low? |  |  | |  | |  |
|  | 4.5 Is the statistical analysis appropriate to answer the research question? |  |  | |  | |  |
| 1. Mixed Methods | 5.1 Is there an adequate rationale for using a mixed methods design to address the research question? |  |  | |  | |  |
|  | 5.2 Are the different components of the study effectively integrated to answer the research question? |  |  | |  | |  |
|  | 5.3 Are the outputs of the integration of qualitative and quantitative components adequately interpreted? |  |  | |  | |  |
|  | 5.4 Are divergencies and inconsistencies between quantitative and qualitative results adequately addressed? |  |  | |  | |  |
|  | 5.5 Do the different components of the study adhere to the quality criteria of each tradition of the methods involved? |  |  | |  | |  |
