# Supplementary material for: Distinguishing Common Digital Phenotyping and Self-Report Parameters for Monitoring and Predicting Depression: Scoping Review
Source: JMIR Mhealth Uhealth. 2026 Mar 2;14:e70840. doi: 10.2196/70840 (PMC12954677; doi:10.2196/70840)
Supplement: Multimedia Appendix 4 [file mhealth-v14-e70840-s004.docx]

Appendix 4

**Detailed quality assessment parameters of included studies**

| **Study id** | **Justification sample size Yes / No** | **n** | **% females** | **Recruitment strategies explained**  **Yes / No** | **Non-participation rates** | **adherence** | **Follow up**  **Yes / No** | **D & B I score** | **MMAT score** |
| --- | --- | --- | --- | --- | --- | --- | --- | --- | --- |
| **1** | Yes | 384, depression: 313 | 88 | Yes | n.a.* | 83.35 | No | 19 | 7 |
| **2** | Yes | S1: 300,  S2: 100 | S1: 40.5  S2: 36.6 | No | N=29 | n.a. | No | 15 | 7 |
| **3** | No | 290 | 64 | No | N=6 | 83.7% | No | 20 | 7 |
| **4** | No | 1178, depression: 92 | n.a. | No | 14.6% of days with data coverage below cut-off | n.a. | No | 12 | 6 |
| **5** | No | 81.277 | n.a. | No | n.a. | 32.3% of initial participants returning for 3rd session | No | 12 | 5 |
| **6** | Yes | 934 | 64 | Yes | n.a. | 77.3% | No | 14 | 7 |
| **7** | No | 65 | 54.5 | No | Cut-off inclusion 20% missing data | n.a. | Yes | 16 | 7 |
| **8** | Yes | 60 | n.a. | No | N=5 | 85.5% | No | 18 | 6 |
| **9** | No | 133, depression: 28 | 74 | Yes | N=2 | 93.32% | No | 23 | 6 |
| **10** | No | 614 | 75.7 | Yes | n.a. | Phone-Active 54.6%, Phone-Passive: 47.7%, Fitbit-Passive: 67.6% | No | 19 | 7 |
| **11** | No | 1 | 0 | No | N=0 | 100% | No | 9 | 6 |
| **12** | No | 18 | 61.1 | Yes | n.a. | n.a. | No | 19 | 6 |
| **13** | Yes | 40, depression: 20 | 85 | No | N=0 | 100% | No | 19 | 7 |
| **14** | No | 623 | 75.6 | No | n.a. | n.a. | No | 15 | 7 |
| **15** | No | 127, depression: 107 | 34 | Yes | n.a. | 70.1% | No | 13 | 6 |
| **16** | No | 173, depression: 43 | 76 | Yes | n.a. | 97.11% | No | 12 | 7 |
| **17** | No | 48 | 76.2 | Yes | N=17 | 64.6% | No | 11 | 5 |
| **18** | No | 315, depression: 136 | 83.8 | Yes | N=154 before T2, N=45 between T2 and T3 | 60.2% | Yes | 22 | 5 |
| **19** |  | 124 | 100 |  |  |  |  |  | 6 |
