# Supplementary material for: Distinguishing Common Digital Phenotyping and Self-Report Parameters for Monitoring and Predicting Depression: Scoping Review
Source: JMIR Mhealth Uhealth. 2026 Mar 2;14:e70840. doi: 10.2196/70840 (PMC12954677; doi:10.2196/70840)
Supplement: Checklist 1 [file mhealth-v14-e70840-s005.docx]

**PRISMA-S Checklist**

| **Section/topic** | **#** | **Checklist item** | **Location(s) Reported** |
| --- | --- | --- | --- |
| **INFORMATION SOURCES AND METHODS** | | | |
| Database name | 1 | Name each individual database searched, stating the platform for each. | We conducted a comprehensive literature search across four electronic databases: PubMed (NCBI), EMBASE, Cochrane Library, Web of Science CC |
| Multi-database searching | 2 | If databases were searched simultaneously on a single platform, state the name of the platform, listing all of the databases searched. | No databases were searched simultaneously on a single platform. All databases (PubMed, EMBASE, Cochrane Library, Web of Science CC) |
| Study registries | 3 | List any study registries searched. | The following study registries were searched for this review: ClinicalTrials.gov, WHO ICTRP, categorized under “Others” in the PRISMA flow chart |
| Online resources and browsing | 4 | Describe any online or print source purposefully searched or browsed (e.g., tables of contents, print conference proceedings, web sites), and how this was done. | No online or print sources were purposefully searched or browsed outside of the four electronic databases. No table of contents, conference proceedings, organizational websites, or print materials were hand-searched for this review. |
| Citation searching | 5 | Indicate whether cited references or citing references were examined, and describe any methods used for locating cited/citing references (e.g., browsing reference lists, using a citation index, setting up email alerts for references citing included studies). | Reference lists of included articles were manually screened to identify additional potentially eligible studies. No citation index or alerting service was used to identify additional studies. |
| Contacts | 6 | Indicate whether additional studies or data were sought by contacting authors, experts, manufacturers, or others. | No additional studies or data were sought by contacting study authors, experts, manufacturers, or others |
| Other methods | 7 | Describe any additional information sources or search methods used. | Selected studies were handsearched and categorized under “Others” in the PRISMA flow chart (no studies retrieved). |
| **SEARCH STRATEGIES** | | | |
| Full search strategies | 8 | Include the search strategies for each database and information source, copied and pasted exactly as run. | **Database:** PubMed (NCBI) **Platform:** NCBI **Date range searched:** January 1, 2021 – November 26, 2025 **Languages:** English, German **Searches were run separately.** **Search mode:** MeSH terms + free-text keywords **Date of final search:** November 26, 2025  **Search 1**  ("Depressive Disorder"[MeSH Terms] OR "Depression"[Title/Abstract])  AND ("Digital Technology"[MeSH Terms] OR "digital health intervention"[Title/Abstract])  **Filters applied:** Publication dates 2021/01/01–2025/11/26; English, German  **Search 2**  ("Depressive Disorder"[MeSH Terms] OR "Depression"[Title/Abstract])  AND ("Digital Technology"[MeSH Terms] OR "digital phenotyping"[Title/Abstract])  **Filters applied:** Publication dates 2021/01/01–2025/11/26; English, German  **Search 3**  ("Self-Management"[MeSH Terms] OR "self-management"[Title/Abstract]  OR "Depression"[Title/Abstract])  AND ("digital phenotyping"[Title/Abstract] OR "Digital Technology"[MeSH Terms])  **Filters applied:** Publication dates 2021/01/01–2025/11/26; English, German  **Search 4**  ("Depressive Disorder"[MeSH Terms] OR "Depression"[Title/Abstract])  AND ("digital phenotyping"[Title/Abstract])  AND ("passive intervention"[Title/Abstract] OR "passive sensing"[Title/Abstract])  **Filters applied:** Publication dates 2021/01/01–2025/11/26; English, German  **Search 5**  ("Depressive Disorder"[MeSH Terms] OR "Depression"[Title/Abstract])  AND ("sensor data"[Title/Abstract] OR "Sensors"[MeSH Terms])  **Filters applied:** Publication dates 2021/01/01–2025/11/26; English  **Database:** EMBASE **Platform:** Elsevier (EMBASE.com) **Date range searched:** January 1, 2021 – November 26, 2025 **Languages:** English, German **Searches run separately** **Fields used:** Title/Abstract (ti, ab), Emtree terms (’/exp’) **Date of final search:** November 26, 2025  **Search 1**  'depression'/exp OR depression:ti,ab)  AND ('digital health intervention':ti,ab OR 'mobile health'/exp OR 'digital health'/exp)  **Filters applied:** Publication year: 2021–2025, Language: English, German  **Search 2**  ('depression'/exp OR depression:ti,ab)  AND ('digital phenotyping':ti,ab)  **Filters applied:** Publication year: 2021–2025, Language: English, German  **Search 3**  ('self management'/exp OR 'self-management':ti,ab OR depression:ti,ab)  AND ('digital phenotyping':ti,ab)  **Filters applied:** Publication year: 2021–2025, Language: English, German  **Search 4**  ('depression'/exp OR depression:ti,ab)  AND ('digital phenotyping':ti,ab)  AND (passive:ti,ab)  **Filters applied:** Publication year: 2021–2025, Language: English, German  **Search 5**  ('depression'/exp OR depression:ti,ab)  AND ('sensor'/exp OR sensor:ti,ab OR 'sensor data':ti,ab)  **Filters applied:** Publication year: 2021–2025, Language: English, German  **Database:** Cochrane Library **Platform:** Wiley Cochrane Library **Date range searched:** January 1, 2021 – November 26, 2025 **Languages:** English (filter applied), German **Search fields used:** Title, Abstract, Keywords **Searches run separately** **Date of final search:** November 26, 2025  **Search 1**  ("Depression":ti,ab,kw OR [mh "Depressive Disorder"])  AND ("digital health intervention":ti,ab,kw)  **Filters applied:** Date: 2021–2025, Language: English, German  **Search 2**  ("Depression":ti,ab,kw OR [mh "Depressive Disorder"])  AND ("digital phenotyping":ti,ab,kw)  **Filters applied:** Date: 2021–2025, Language: English, German  **Search 3**  ("self-management":ti,ab,kw OR [mh "Self Care"])  AND ("digital phenotyping":ti,ab,kw)  **Filters applied:** Date: 2021–2025, Language: English, German  **Search 4**  ("Depression":ti,ab,kw OR [mh "Depressive Disorder"])  AND ("digital phenotyping":ti,ab,kw)  AND (passive:ti,ab,kw)  **Filters applied:** Date: 2021–2025, Language: English, German  **Search 5**  ("Depression":ti,ab,kw OR [mh "Depressive Disorder"])  AND (sensor:ti,ab,kw OR sensors:ti,ab,kw OR [mh "Sensors"])  **Filters applied:** Date: 2021–2025, Language: English, German  **Database:** Web of Science Core Collection (Science Citation Index Expanded, Social Sciences Citation Index, Emerging Sources Citation Index) **Platform:** Clarivate Web of Science **Search field:** **TS = Topic** (Title, Abstract, Author Keywords, Keywords Plus) **Date range searched:** January 1, 2021 – November 26, 2025 **Language:** English, German **Searches run separately** **Date of final search:** November 26, 2025  **Search 1**  TS=(Depression AND "digital health intervention")  **Filters applied:** Timespan: 2021–2025, Languages: English, German  **Search 2**  TS=(Depression AND "digital phenotyping")  **Filters applied:** Timespan: 2021–2025, Languages: English, German  **Search 3**  TS=(("self-management" OR Depression) AND "digital phenotyping")  **Filters applied:** Timespan: 2021–2025, Languages: English, German  **Search 4**  TS=(Depression AND "digital phenotyping" AND passive)  **Filters applied:** Timespan: 2021–2025, Languages: English, German  **Search 5**  TS=(Depression AND ("sensor data" OR sensor OR sensors))  **Filters applied:** Timespan: 2021–2025, Languages: English, German  **Registry:** ClinicalTrials.gov **Platform:** U.S. National Library of Medicine (NLM) **Date range searched:** January 1, 2021 – November 26, 2025 **Languages:** No language filter available (default = all languages) **Searches run separately Search mode:** Condition / Other terms fields **Date of final search:** November 26, 2025  **Search 1**  Depression AND "digital health intervention"  **Filters applied:** Study start date: From 01/01/2021, Recruitment: All statuses  **Search 2**  Depression AND "digital phenotyping"  **Filters applied:** Study start date ≥ 01/01/2021  **Search 3**  ("self-management" OR Depression) AND "digital phenotyping"  **Filters applied:** Study start date ≥ 01/01/2021  **Search 4**  Depression AND "digital phenotyping" AND passive  **Filters applied:** Study start date ≥ 01/01/2021  **Registry:** WHO International Clinical Trials Registry Platform (ICTRP) **Platform:** ICTRP Search Portal **Date range searched:** January 1, 2021 – November 26, 2025 **Languages:** All languages **Searches run separately Date of final search:** November 26, 2025  **Search 1**  Depression digital health intervention  **Filters applied:** Recruitment status: All, Study phase: All, Study type: All, Registration date ≥ 01/01/2021  **Search 2**  Depression digital phenotyping  **Filters:** Registration date ≥ 01/01/2021  **Search 3**  self-management digital phenotyping  **Filters:** Registration date ≥ 01/01/2021  **Search 4**  Depression passive digital phenotyping  **Filters:** Registration date ≥ 01/01/2021  **Search 5**  Depression sensor  **Filters:** Registration date ≥ 01/01/2021  **Search 5**  Depression AND sensor  **Filters applied:** Study start date ≥ 01/01/2021 |
| Limits and restrictions | 9 | Specify that no limits were used, or describe any limits or restrictions applied to a search (e.g., date or time period, language, study design) and provide justification for their use. | Our searches applied limits for publication date and language. Specifically, records were restricted to studies published between January 1, 2021 and November 26, 2025, and to publications in English or German. These limits were used to ensure that the review captured the most current developments in digital interventions and digital phenotyping, as the technological landscape in this field evolves rapidly. No additional limits (e.g., study design filters, human subject filters, population filters) were applied. |
| Search filters | 10 | Indicate whether published search filters were used (as originally designed or modified), and if so, cite the filter(s) used. | No published search filters were used for this review. All search strategies were developed manually using combinations of keywords and MeSH terms relevant to depression, digital phenotyping, and digital health interventions. The search strategy did not employ any existing validated filters (eg, clinical study filters or methodological filters). |
| Prior work | 11 | Indicate when search strategies from other literature reviews were adapted or reused for a substantive part or all of the search, citing the previous review(s). | No search strategies from previous literature reviews were adapted or reused. All search strings were developed specifically for this review based on the research objectives and inclusion criteria. |
| Updates | 12 | Report the methods used to update the search(es) (e.g., rerunning searches, email alerts). | The searches were updated manually by rerunning all database queries prior to final analysis. No automated alerts, citation tracking tools, or continuous updating services were used. |
| Dates of searches | 13 | For each search strategy, provide the date when the last search occurred. | All searches were last conducted on November 26, 2025 across all included databases. |
| **PEER REVIEW** | | | |
| Peer review | 14 | Describe any search peer review process. | No formal search peer review process was conducted for this review. |
| **MANAGING RECORDS** | | | |
| Total Records | 15 | Document the total number of records identified from each database and other information sources. | The total number of records identified from each database and other information sources was 3,243 (PubMed n=518, Cochrane Library n=1256, EMBASE n=791, Web of Science n=276, Others n=402) |
| Deduplication | 16 | Describe the processes and any software used to deduplicate records from multiple database searches and other information sources. | Records retrieved from all databases and clinical trial registries were exported into Microsoft Excel and manually reviewed to identify and remove duplicates. Deduplication was performed by comparing study titles, authors, publication years, and digital object identifiers (DOIs). No automated or software-assisted deduplication tools (e.g., Covidence, EndNote) were used. |
|  |  |  |  |
| PRISMA-S: An Extension to the PRISMA Statement for Reporting Literature Searches in Systematic Reviews | | |  |
| Rethlefsen ML, Kirtley S, Waffenschmidt S, Ayala AP, Moher D, Page MJ, Koffel JB, PRISMA-S Group. | | |  |
| Last updated February 27, 2020. | |  |  |
